# Elucidation of Teicoplanin Interactions with Drug Targets Related to COVID-19

**DOI:** 10.3390/antibiotics10070856

**Published:** 2021-07-15

**Authors:** Faizul Azam

**Affiliations:** Department of Pharmaceutical Chemistry & Pharmacognosy, Unaizah College of Pharmacy, Qassim University, Unaizah 51911, Saudi Arabia; faizulazam@gmail.com or f.azam@qu.edu.sa; Tel.: +966-50-2728652

**Keywords:** SARS-CoV-2, teicoplanin, docking, molecular dynamics, MM/GBSA

## Abstract

Teicoplanin is a glycopeptide antibiotic effective against several bacterial infections, has exhibited promising therapeutic efficiency against COVID-19 in vitro*,* and the rationale for its use in COVID-19 is yet to be recognized. Hence, in this study a number of molecular modeling techniques were employed to decrypt the mechanistic insight of teicoplanin interaction with several COVID-19 drug targets. Initially, molecular docking was employed to study the teicoplanin interaction with twenty-five SARS-CoV-2 structural and non-structural proteins which was followed by molecular mechanics/generalized Born surface area (MM/GBSA) computation for binding energy predictions of top ten models from each target. Amongst all macromolecular targets, the N-terminal domain of the nucleocapsid protein displayed the strongest affinity with teicoplanin showing binding energies of −7.4 and −102.13 kcal/mol, in docking and Prime MM/GBSA, respectively. Thermodynamic stability of the teicoplanin-nucleocapsid protein was further probed by molecular dynamics simulations of protein–ligand complex as well as unbounded protein in 100 ns trajectories. Post-simulation MM-GBSA computation of 50 frames extracted from simulated trajectories estimated an average binding energy of −62.52 ± 12.22 kcal/mol. In addition, conformational state of protein in complex with docked teicoplanin displayed stable root-mean-square deviation/fluctuation. In conclusion, computational investigation of the potential targets of COVID-19 and their interaction mechanism with teicoplanin can guide the design of novel therapeutic armamentarium for the treatment of SARS-CoV-2 infection. However, additional studies are warranted to establish the clinical use or relapses, if any, of teicoplanin in the therapeutic management of COVID-19 patients.

## 1. Introduction

The outbreak of COVID-19 pandemic in China, caused by severe acute respiratory syndrome coronavirus-2 (SARS-CoV-2) primarily spreads through close contact amongst people by sneezing, coughing, or by communicating verbally. The primary symptoms of the disease are fever, cough, fatigue, shortness of breath, and loss of smell. The complications of this disease include viral pneumonias, respiratory distress, and hypoxia [1]. The world is suffering due to lack of effective medicine. Therefore, there is an urgent need to figure out efficient medicine through drug discovery efforts [2,3].

**Figure 1:**
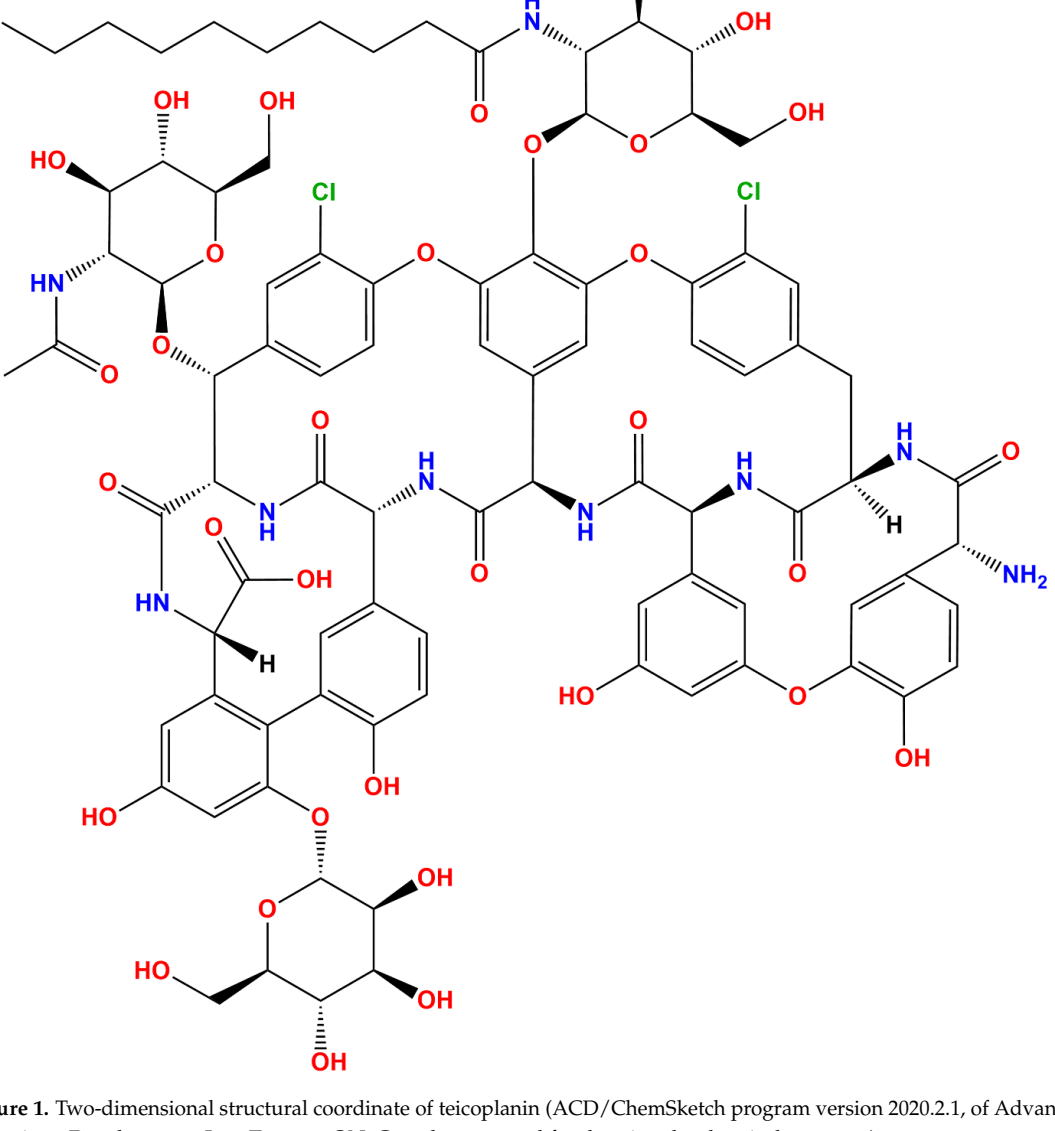
Two-dimensional structural coordinate of teicoplanin (ACD/ChemSketch program version 2020.2.1, of Advanced Chemistry Development, Inc., Toronto, ON, Canada, was used for drawing the chemical structure).

Drug repurposing strategy is often used for recognizing novel usages of approved or investigational drugs because of several advantages such as lower risk of failure, reduced time for drug development, and lower investment needs [4]. Therefore, numerous antimalarials, antibacterials, antiparasitic agents, and antivirals are currently prevalent in pre-clinical as well as clinical investigations aimed at developing COVID-19 treatment by a drug repurposing approach [5]. In particular, a widely available FDA-approved antibiotic, teicoplanin (Figure 1), is among the molecule of interest as probable COVID-19 medicine. It belongs to the glycopeptide class of antibiotic having low toxicity profile in humans and hence routinely used in clinical practice for the treatment of bacterial infections. Interestingly, it has demonstrated antiviral efficacy against several kinds of viruses such as Ebola, MERS-CoV, and SARS-CoV [6]. Very recently, it has been reported that teicoplanin can thwart the cellular entry of SARS-CoV-2 at an impressive 1.66-μM concentration [7,8]. Furthermore, IC_50_ value of 1.5 μM was recorded for inhibition of the SARS-CoV-2 main protease by teicoplanin [9].

Computer-aided drug design methods are extensively employed in drug design and discovery projects owing to several advantages such as rapid development process and reduced cost [10,11,12]. In particular, molecular docking coupled with molecular dynamics simulation studies are intended to decipher the mechanism of binding interactions at the molecular levels [13]. Rapid mechanistic insight is vital for understanding structure–activity relationship and leading to the optimization of the design and discovery of potential molecules [14,15,16]. In this study, several computational techniques such as molecular docking, molecular mechanics/generalized Born surface area (MM-GBSA), and molecular dynamics simulation were exploited to inspect the binding interactions between teicoplanin and potential drug targets associated with SARS-CoV-2. The study is envisioned to assist in finding potential leads and accelerating drug development process for the treatment of novel coronavirus, COVID-19.

## 2. Experimental Section

**Figure 2:**
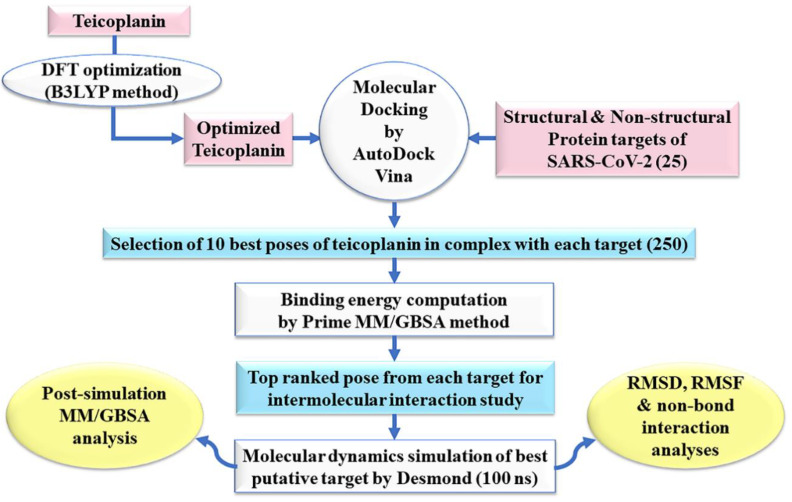
An outline of the adopted methodology in this study. B3LYP: Becke’s three-parameter hybrid model, Lee–Yang–Parr correlation functional method; DFT: density functional theory; MM/GBSA: molecular mechanics/generalized Born surface area; ns: nano seconds; RMSD: root-mean-square deviation; RMSF: root-mean-square fluctuation.

The methodology adopted in this study has been outlined as flowchart in Figure 2.

### 2.1. Ligand Preparation

**Figure 3:**
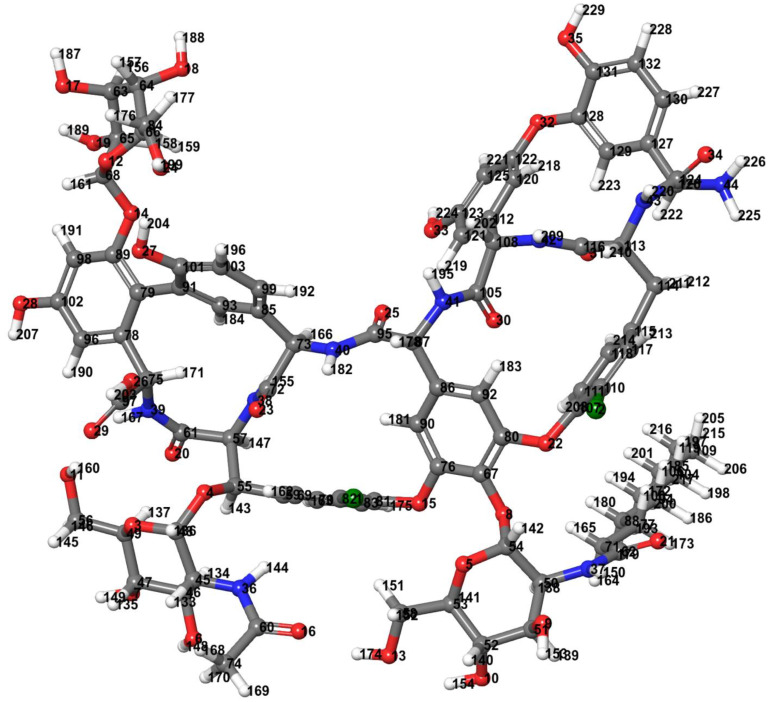
Teicoplanin structure optimized by density functional theory (DFT) with Becke’s three-parameter hybrid model, the Lee–Yang–Parr (B3LYP) correlation functional method at 6-31G level (Jaguar v10.9 software of Schrodinger Suites 2020-3 was used). Color code: carbon—dark gray, hydrogen—light gray, nitrogen—blue, oxygen—red, and chlorine—green.

The molecular structure of teicoplanin was retrieved from PubChem database as two-dimensional coordinate in sdf format and converted to its three-dimensional conformation by means of Open Babel program [17]. Jaguar v10.9 of Schrodinger Suites 2020-3 was used for density functional theory (DFT)-based optimization by Becke’s three-parameter hybrid model, the Lee–Yang–Parr (B3LYP) method at the level of 6-31G [18,19]. The optimized structure of teicoplanin is presented in Figure 3. Prepare_ligand4.py module of MGL Tools 1.5.6 [20] was used for the preparation of ligand in pdbqt format after merging all non-polar hydrogens, defining rotatable bonds/torsion tree and adding Gasteiger charges.

### 2.2. Protein Preparation

Twenty-five structural and non-structural protein targets related to SARS-CoV-2 were downloaded from the RCSB PDB repository as listed in Table 1. The macromolecular targets include main protease (M^Pro^), papain-like protease (PL^Pro^), RNA-dependent RNA polymerase (RdRp-RTP site), RdRp-RNA site, spike protein-receptor binding domain (RBD), spike trimer (open state), spike monomer (closed state), S2 protein in post-fusion state, N-protein (C-domain), N-protein (N-domain), Nsp3-AMP site, Nsp3-MES site, Nsp7, Nsp8, Nsp9, Nsp10, Nsp12, helicase (Nsp13-ADP site), helicase (Nsp13-NCB site), Nsp14 (N-terminal exoribonuclease; ExoN), Nsp14 (N7 methyltransferase; N7-MTase), Nsp15 (exoribonuclease), Nsp16 (GTA site), Nsp16 (MGP site), and Nsp16 (SAM site). All the protein structures were processed in Biovia Discovery Studio Visualizer 2020 [21] and PyMol 2.4.1 [22] for removing unwanted co-crystallized compounds including water molecules. MGLTools 1.5.6 [20] was utilized for generating input receptor files in pdbqt format. 

### 2.3. Molecular Docking Simulation

The grid box of 30 × 30 × 30 size in x, y, and z directions with 1-Å spacing was constructed around the active site residues as demarcated by the bound co-crystallized ligands (see Table 1 for grid points used for each receptor). However, blind docking, covering the entire macromolecular target, was opted if binding site information was unavailable. AutoDock Vina 1.1.2 [23] was used for molecular docking employing default parameters for flexible ligand and rigid protein. Moreover, the exhaustiveness parameter was set to 12. Upon successful completion of docking simulation, ten best poses were individually analyzed for intermolecular interactions using PyMol 2.4.1 [22] and Biovia Discovery Studio Visualizer 2020 [16,21,24] and further subjected to MM/GBSA computations in the next step.

### 2.4. Prime MM-GBSA Calculations

MM-GBSA technique are frequently used to rationalize the findings of molecular docking and virtual screening. Prime MM-GBSA module of Schrödinger Suite 2020-3 [25,26,27] was used to compute binding energy and relevant parameters as presented in Table 2. The Prime MM-GBSA protocol amalgamates OPLS molecular mechanics energies, a VSGB solvation model for polar solvation (G_SGB_), and a nonpolar solvation expression (G_NP_) involving nonpolar solvent-accessible surface area (SASA) and van der Waals interactions [28]. The binding-free energy (Δ*G*_bind_) of docked teicoplanin in complex with respective proteins was calculated using the following equation [29]:

Δ*G*_bind_ = Δ*E*_MM_ + Δ*G*_solv_ + Δ*G*_S_ (1)

where Δ*E*_MM_ is the difference in energy between the complex structure and the sum of the energies of the protein with and without teicoplanin, Δ*G*_solv_ is the difference in the GBSA solvation energy of the teicoplanin–protein complex and the sum of the solvation energies for the teicoplanin-bound and unbound protein, and Δ*G*_SA_ is the difference in the energy of surface area for the teicoplanin–protein complex and the sum of the surface area energies for the ligand and un-complexed protein.

### 2.5. Molecular Dynamics Simulation

The best ranked conformation of teicoplanin in complex with N-protein–N-domain furnished by MM-GBSA experiments was further assessed for their thermodynamic behavior and stability by employing molecular dynamics (MD) simulation. Desmond 6.1 program integrated with Maestro (Schrödinger, Inc. LLC, New York, NY, USA) was used for MD simulation studies [30,31]. In addition, apo form of the N-protein–N-domain was also simulated in order to understand the conformational changes upon teicoplanin binding. The teicoplanin–protein complex was placed into an orthorhombic box of 10 × 10 × 10 Å size, filled with 7210 water molecules by means of simple point charge (SPC) model implemented in system setup protocol. An OPLS3 force field was applied for the MD computations [32]. The system was neutralized using 20 and 26 Na^+^ and Cl^−^ ions, respectively, with a salt concentration of 0.15 M that represents the physiological concentration of monovalent ions. An isothermal–isobaric (NPT) ensemble was utilized with temperature and pressure attuned to 300 K and 1.01325 bar, respectively. A simulation time of 100 ns was adjusted, whereas trajectories were saved at every 100 ps. A cut-off radius of 9.0 Å was used for short-range van der Waals and Coulomb interactions. Nose–Hoover thermostat [33] and Martyna–Tobias–Klein [34] methods were employed for maintaining the system temperature and pressure, respectively. In order to integrate the equations of motion, an RESPA integrator was used with an inner time step of 2.0 fs for bonded as well as non-bonded interactions within the short-range cut-off [35]. The system was minimized and equilibrated with the default protocols of the Desmond. Simulation event analysis, simulation quality analysis, and simulation interaction diagram protocols of the Desmond package was exercised to analyze the trajectory files.

### 2.6. Post-Simulation MM-GBSA Analysis

Post-simulation MM-GBSA analysis was performed by using the thermal_MMGBSA.py script of the Prime/Desmond module of the Schrodinger suite 2020-3 [25,26]. From each MD trajectory, every tenth frame was extracted from the last 50 ns of simulated trajectory, averaging over 50 frames, for binding free energy calculations of teicoplanin in complex with N-protein–N-domain. The Prime MM-GBSA method uses the rule of additivity wherein total binding free energy (kcal/mol) represents a summation of individual energy modules like coulombic, covalent, hydrogen bond, van der Waals, self-contact, lipophilic, solvation, and π–π stackings of ligand and protein [36].

## 3. Results and Discussion

### 3.1. Validation of Docking Protocol

The co-crystallized ligand of main protease, N3, was redocked into the inhibitor binding cavity of the enzyme and docked conformation of the ligand was compared with that of the crystal structure in order to validate the docking protocol implemented in AutoDock Vina. The root-mean-square deviation (RMSD) was less than 2 Å between the docked and X-ray crystal structure conformations of N3, which authenticated the scoring functions implemented in the docking program (data not shown). According to the reported protocols, a successful docking computation should furnish the RMSD of ≤2.0 Å [10,14]. Therefore, AutoDock Vina used in this study was deemed reliable for studying teicoplanin interaction with potential SARS-CoV-2 targets.

### 3.2. Molecular Docking of Teicoplanin with Potential Targets of SARS-CoV-2

Molecular docking is a computational method of accelerating the early stages of drug discovery through unravelling the precise mechanism of intermolecular interactions of potential drug candidates by comparing their energetic compatibility and molecular shape with the target proteins [11,37]. With the aim of inspecting the interaction of teicoplanin with putative drug targets of SARS-CoV-2, docking studies was performed on the receptors/targets constituting both non-structural and structural proteins with prospective to be used in the drug discovery field [38,39]. Docking-predicted binding energies of teicoplanin with twenty-five COVID-19 drug targets are given in Table 1, whereas comprehensive intermolecular interactions have been listed in Table S1 in the Supplementary Information.

### 3.3. Prime MM-GBSA Calculations of Docked Complexes

Though several docking experiments are routinely employed to emphasize the binding mode and the affinity of a ligand relative to the protein, lack of accurate scoring function is one of the drawbacks of these algorithms. Therefore, MM-GBSA computations are routinely employed as a post-docking analysis in order to avoid false negatives as well as false positives [40,41]. The top ten conformations of protein–teicoplanin-docked complexes of each target were subjected to molecular mechanics/generalized Born surface area (MM-GBSA) computation for estimation of more accurate free binding energies. Table 2 enlists the MM-GBSA computed energies of best conformation of teicoplanin in complex with each target. Several other energy components such as Coulomb energy, hydrogen-bonding correction, lipophilic energy, generalized Born electrostatic solvation energy, Van der Waals energy, and ligand strain energy were also computed. Moreover, an elaborated MM-GBSA calculation of all poses has been listed in Table S2 in the Supplementary Information.

**Figure 4:**
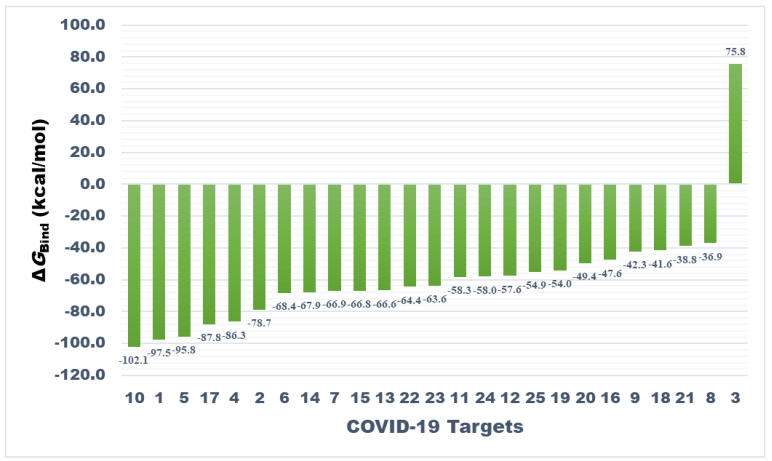
Bar plot showing comparative affinity of teicoplanin against drug targets associated with COVID-19 (please refer to Table 2 for target names against respective numbers). The analysis is based on molecular mechanics–generalized Born surface area (MM-GBSA) computed binding energy (Δ*G*_Bind_) in kcal/mol.

As presented in Figure 4, nucleocapsid protein N-terminal domain ranked top among all studied targets exhibiting Δ*G*_bind_ = −102.13 kcal/mol followed by main protease and spike protein-RBD with binding energies of −97.55 and −95.76 kcal/mol, respectively. However, MM-GBSA binding energy of teicoplanin-main protease complex obtained from AutoDock 4.2 has been recently reported as -68 kcal/mol. Interestingly, RdRp-RTP site was rendered as least promising for teicoplanin in this study, demonstrating 75.8 kcal/mol binding energy. The most promising target also hosted ample non-bond interactions with teicoplanin within the active site of N-domain of the N-protein. Moreover, appreciable intermolecular forces were established with all other targets. It is evident that the N-domain of the N-protein hosts nucleic acid substrates during processing of the viral genome into a ribonucleoprotein unit. Therefore, targeting this protein by teicoplanin can interrupt crucial steps of the SARS-CoV-2 life cycle such as transcription and translation processes [42]. Several proteins such as Nsp12, RdRp-RNA site, and papain-like protease were ranked as moderately favorable targets, whereas variable affinity was noted against the rest of the targets. The chemical structure of teicoplanin represents a unique blend of polar groups in the form of several amino acid fragments, carbohydrate cores such as N-acetylglucosamine, β-D-glucosamine and mannose, and non-polar fragments such as fatty acyl side-chain and numerous aromatic rings. These moieties enabled teicoplanin to interact with diverse drug targets of COVID-19 which warrants further experimental validation and may be useful in drug design based on teicoplanin template. Intermolecular interactions observed after MM-GBSA computation of all studied targets are presented in Figures S1–S4 and Table S1 in the Supplementary Information.

### 3.4. Molecular Dynamics Simulation Studies

**Figure 5:**
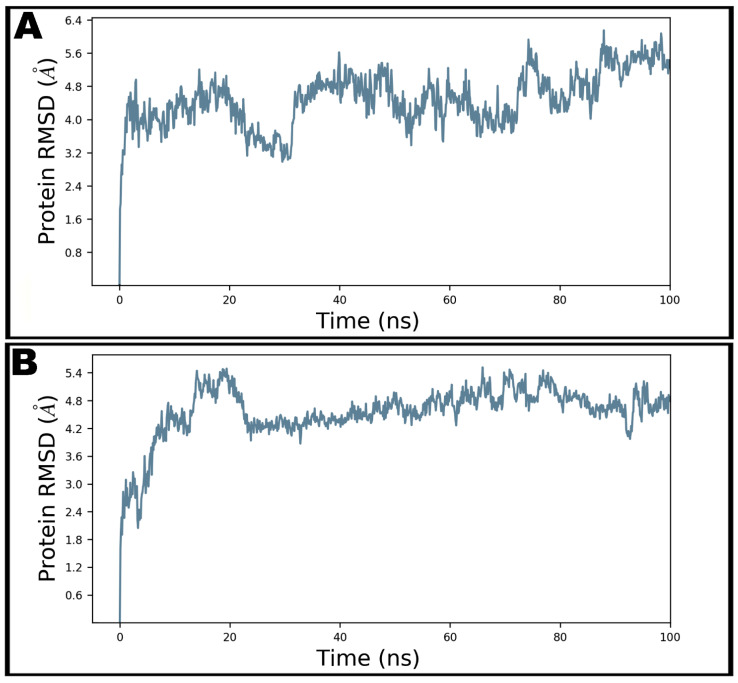
The root-mean-square deviations (RMSD) of Cα atoms of N-protein–N-domain in unbound state (**A**) and teicoplanin-bound state (**B**) during 100 ns molecular dynamics simulation.

**Figure 6:**
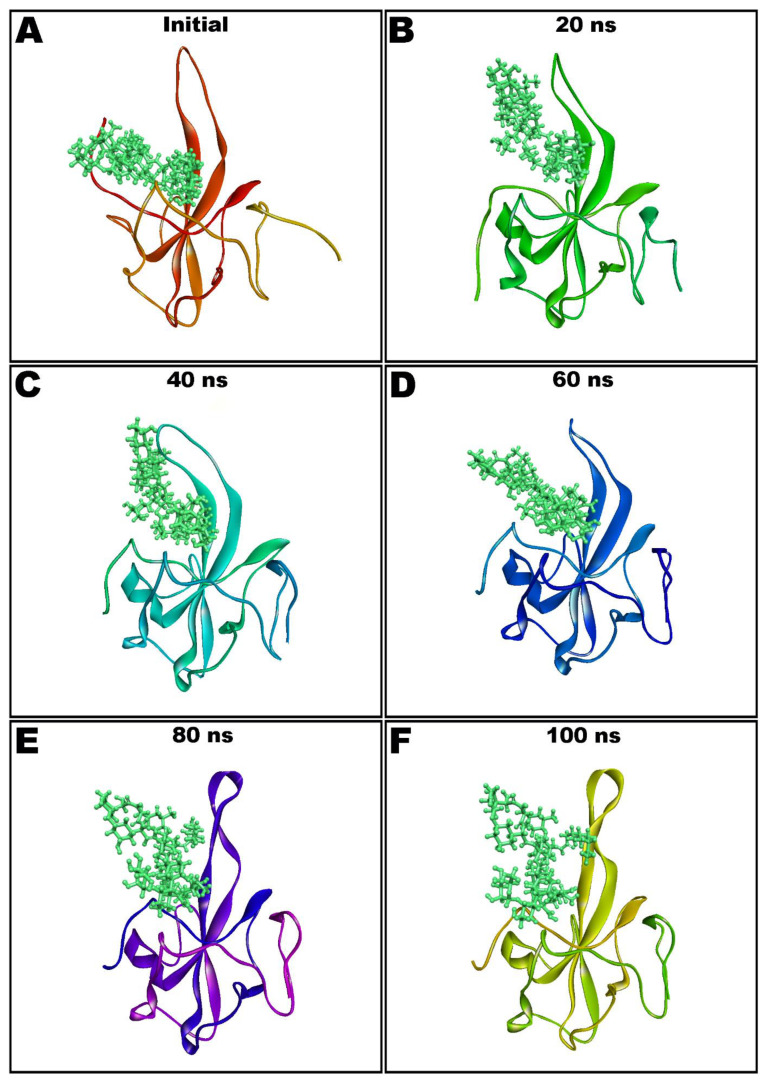
Molecular dynamics (MD) simulation snapshots of teicoplanin (shown as ball and stick style in green color) in complex with nucleocapsid protein N-domain (depicted as solid ribbon) showing conformational stability (Initial: (**A**); after 20 ns: (**B**); 40 ns: (**C**); 60 ns: (**D**); 80 ns: (**E**); and 100 ns: (**F**)).

Dynamic and thermodynamics parameters of living systems under specific conditions of physiological environments can be estimated by the application of molecular dynamics (MD) simulation, a widely employed computer-aided drug design technique [11,13,14,43]. Therefore, the best docked pose of teicoplanin in complex with SARS-CoV-2 nucleocapsid protein N-terminal domain was subjected to MD simulation study in order to investigate the stability of the ligand–protein complex as well as main intermolecular interactions during the simulated trajectory. MM-GBSA-optimized pose bearing minimum binding energy was selected for MD simulation study. Desmond software was employed for the MD simulation of 100 ns in explicit solvent system. The resulting trajectories of the simulated complex was inspected for different standard simulation parameters such as backbone RMSDs for alpha-carbons, side chains, and heavy atoms. In addition, the root-mean-square fluctuations (RMSFs) of individual amino acid residue and intermolecular interactions involved were also evaluated. Figure 5A shows the RMSD of Cα atoms of apo protein whereas the complex of teicoplanin bound to N-protein–N-domain is presented as Figure 5B. The analysis of RMSD of teicoplanin-bound protein indicates that the simulated system fluctuated initially during 0–22 ns but acquired stability during rest of the simulated time. However, fluctuations can be clearly seen in the RMSD plot of the apo protein alone (Figure 5A). Therefore, it seems logical to infer that upon teicoplanin binding, the protein maintains its conformational stability when simulated for 100 ns period. The conformational changes observed during simulated path has been visualized by the snapshots taken at the intervals of every 20 ns and presented in Figure 6.

The MM-GBSA method exploits molecular mechanics, the generalized Born solvation models and a solvent accessibility approach to estimate the free energies of binding based on snapshots obtained from MD simulations [40,44]. MM-GBSA computation was performed for the last 50 snapshots of 100 ns simulation, which estimated an average binding energy of −62.52 ± 12.22 kcal/mol.

**Figure 7:**
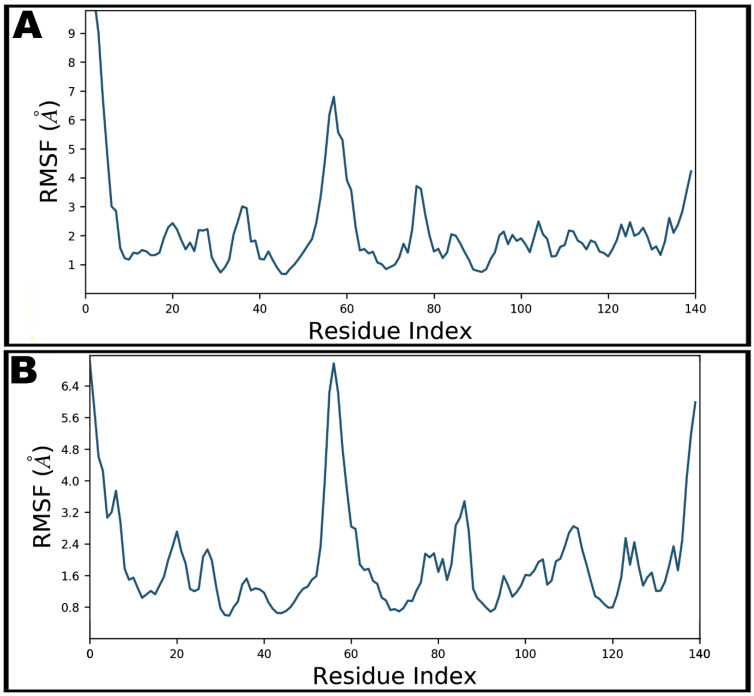
Root-mean-square fluctuations (RMSF) of SARS-CoV-2 nucleocapsid protein N-domain during 100 ns molecular dynamics simulation in apo form (**A**) and teicoplanin bound form (**B**).

The local conformational alterations along SARS-CoV-2 nucleocapsid protein N-domain were investigated by analyzing the RMSF during simulation time. Terminal residues fluctuated most in both bound and unbound states. Maximum fluctuation was noted with Gly1 as 13.29 Å which was declined upon teicoplanin binding at 7.00 Å (Figure 7). Key residues involved in hydrogen bonding showed fluctuations at 3.74, 2.97, 1.76, 1.54, 4.12, 0.96, and 2.68 Å by Asn7, Asn8, Thr9, Ser11, Arg55, Tyr69, and Pro111, respectively.

**Figure 8:**
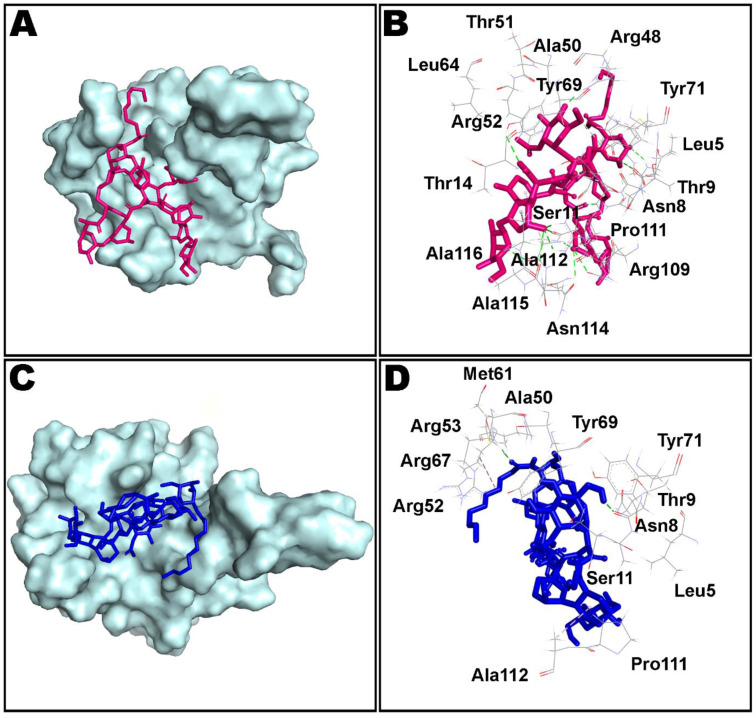
Non-bond interactions observed in first (**A***,***B**) and last (**C**,**D**) frames extracted from 100 ns molecular dynamics simulation of teicoplanin bound nucleocapsid protein N-domain of SARS-CoV-2. Ala: alanine; Arg: arginine; Asn: asparagine; Leu: leucine; Met: methionine; Pro: proline; Ser: serine; Thr: threonine; Tyr: tyrosine. The number of corresponding amino acid residue is in accordance with the crystallographic information obtained from the protein data bank.

**Figure 9:**
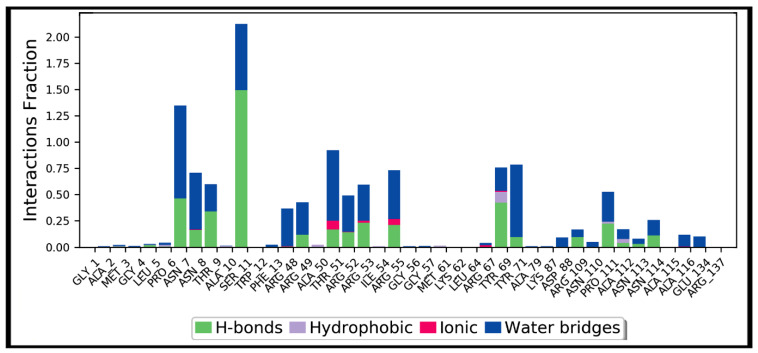
Protein interactions with teicoplanin, monitored throughout the simulation trajectory. These interactions are clustered by type and summarized in bar diagram including hydrogen bonds (H-bonds), hydrophobic, ionic, and water bridges. Ala: alanine; Arg: arginine; Asn: asparagine; Asp: aspartic acid; Cys: cysteine; Gln: glutamine; Glu: glutamic acid; Gly: glycine; His: histidine; Leu: leucine; Met: methionine; Phe: phenylalanine; Pro: proline; Ser: serine; Thr: threonine; Tyr: tyrosine; Val: valine. The number of corresponding amino acid residue is according to the crystallographic information obtained from the protein data bank.

Simulation interactions diagrams presented in Figure 8 and Figure 9 during entire simulation time signifies a comprehensive intermolecular interaction profile of teicoplanin with nucleocapsid protein N-domain. The modus of interaction pattern of teicoplanin illustrates that only fewer docking-predicted main contacts were retained upon MD simulation time of 100 ns. Figure 8 presents intermolecular interactions observed in first and last frames of the teicoplanin bound to nucleocapsid protein N-domain. Residues participating in both hydrogen bonding and water bridges include Asn7, Asn8, Thr9, Ser11, Thr51, Arg53, Arg55, Tyr71, Pro111, and Asn114. In addition, Thr51, Arg53, Arg55, and Arg67 also contributed in ionic bond interaction. However, amino acid residues Ala10, Ala50, Tyr69, and Ala112 were noted to be important for hydrophobic interaction.

## 4. Conclusions

By using computer-aided drug design techniques, the current study explained the intermolecular interaction of the antibacterial drug, teicoplanin, with potential drug targets of SARS-CoV-2. Molecular docking studies employing AutoDock Vina highlighted the importance of hydrophilic and hydrophobic interactions in supporting the teicoplanin molecule inside the nucleocapsid protein N-domain binding cavity. MM-GBSA analysis and molecular dynamics simulation results not only reinforce the credibility of the docking results, but also authenticate the stability of the simulated system. This study is expected to assist lead optimization and design of COVID-19 drugs based on molecular skeleton of teicoplanin. However, the requirement of additional experimental and clinical validation is an obvious limitation of this study. Nevertheless, findings of this study justify further exploration of teicoplanin repurposing.

## Figures and Tables

**Table 1:** Docking-predicted binding energy (Δ*G*_Bind_) and grid parameters used for docking of teicoplanin with 25 potential COVID-19 drug targets.

Target Number	Targets	PDB ID	Grid Center for AutoDock Vina Program			Docking Δ*G*_Bind_ (kcal/mol)
			x	y	z	
1.	Main protease	6LU7	−9.732	11.403	68.925	−5.4
2.	Papain-like protease	6WUU	22.225	68.703	4.704	−5.4
3.	RdRp (RTP site)	7BV2	91.776	91.560	104.863	−9.8
4.	RdRp (RNA site)	7BV2	71.227	92.269	112.852	−7.7
5.	Spike protein (RBD)	6M0J	−36.193	37.260	−5.752	−6.1
6.	Spike monomer	6VXX	219.061	220.947	261.311	−5.4
7.	Spike trimer	6VYB	251.872	195.411	243.040	−6.9
8.	S2 protein (post fusion state)	6LXT	−0.641	11.084	28.359	−5.3
9.	N-protein (C-domain)	6YUN	−10.288	12.683	7.740	−5.6
10.	N-protein (N-domain)	6YI3	16.299	11.628	6.638	−7.4
11.	Nsp3 (AMP site)	6W6Y	9.124	−8.677	16.220	−7.3
12.	Nsp3 (MES site)	6W6Y	23.830	9.255	54.812	−5.9
13.	Nsp7	7BV2	104.786	80.343	127.861	−4.4
14.	Nsp8	7BV2	108.168	116.454	120.901	−5.9
15.	Nsp9	6WXD	53.119	−10.095	22.482	−4.5
16.	Nsp10	6WVN	64.644	15.650	9.522	−2.2
17.	Nsp12	7BV2	97.382	97.966	93.920	−7.8
18.	Nsp13 (helicase ADP site)	6JYT	405.020	47.480	62.350	−6.5
19.	Nsp13 (helicase NCB site)	6JYT	423.816	33.797	56.132	−5.4
20.	Nsp14 (ExoN)	5C8S	−39.712	−50.654	15.5594	−7.1
21.	Nsp14 (N7mtase)	5C8S	−10.273	−42.259	−7.644	−3.0
22.	Nsp15 (Exoribonuclease)	6WLC	94.134	−19.803	−25.857	−6.5
23.	Nsp16 (GTA site)	6WVN	84.158	24.757	37.836	−6.2
24.	Nsp16 (MGP site)	6WVN	100.029	38.995	18.481	−7.4
25.	Nsp16 (SAM site)	6WVN	84.156	15.450	26.991	−6.5

ADP site: adenosine diphosphate binding site; AMP: adenosine monophosphate; C-domain: carbon-terminal domain; ExoN: N-terminal exoribonuclease; GTA: P1-7-methylguanosine-P3-adenosine-5′,5′-triphosphate; MES: 2-(N-morpholino)-ethanesulfonic acid; MGP: 7-methyl-guanosine-5′-triphosphate; N-domain: nitrogen-terminal domain; N7mtase: N7 methyltransferase; NCB site: nucleic acids binding site; N-protein: nucleocapsid protein; Nsp: non-structural protein; PDB: protein data bank; RBD: receptor binding domain; RdRp: RNA-dependent RNA polymerase; RNA: ribonucleic acid; RTP: remdesivir; SAM: S-adenosylmethionine.

**Table 2:** Results of molecular mechanics/generalized Born surface area (MM/GBSA) computations for teicoplanin in complex with 25 potential COVID-19 targets.

Target Number	Target	Δ*G*_Bind_^a^	Δ*G*_Coul_^b^	Δ*G*_HBond_^c^	Δ*G*_Lipo_^d^	SolvGB ^e^	Δ*G*_vdw_^f^	Lig SE ^g^
1.	Main protease	−97.55	−19.11	−0.68	−59.81	44.25	−68.46	3.87
2.	Papain-like protease	−78.75	−39.06	−3.33	−58.73	54.34	−42.74	39.85
3.	RdRp (RTP site)	75.76	−124.58	−4.94	−91.90	209.45	−17.65	184.45
4.	RdRp (RNA site)	−86.33	−68.97	−6.24	−13.97	78.93	−83.45	10.54
5.	Spike protein (RBD)	−95.76	−32.67	−2.13	−54.25	48.60	−66.71	18.19
6.	Spike monomer	−68.38	−61.51	−4.39	−13.35	55.98	−50.51	12.74
7.	Spike trimer	−66.87	−27.37	−4.11	−21.53	53.95	−65.20	11.90
8.	S2 protein (post fusion state)	−36.93	−23.22	−2.74	−14.11	55.19	−61.27	24.45
9.	N-protein (C-domain)	−42.31	−30.82	−5.06	−16.05	64.88	−62.43	26.93
10.	N-protein (N-domain)	−102.13	−40.95	−6.98	−24.95	61.07	−78.69	−0.90
11.	Nsp3 (AMP site)	−58.33	−42.82	−4.38	−25.86	64.42	−71.09	32.93
12.	Nsp3 (MES site)	−57.60	−12.61	−2.42	−30.13	34.62	−75.40	37.34
13.	Nsp7	−66.61	−54.39	−4.15	−19.17	55.49	−60.78	8.43
14.	Nsp8	−67.93	−10.55	−2.28	−25.51	28.75	−68.85	16.42
15.	Nsp9	−66.83	−43.87	−3.59	−19.55	46.75	−57.02	10.15
16.	Nsp10	−47.56	−29.62	−2.47	−23.97	57.87	−62.53	40.47
17.	Nsp12	−87.84	−96.33	−8.48	−19.26	135.75	−108.20	8.52
18.	Nsp13 (helicase ADP site)	−41.57	−63.94	−7.00	−19.36	88.70	−53.79	16.23
19.	Nsp13 (helicase NCB site)	−53.99	−42.21	−4.05	−26.40	87.73	−78.57	7.39
20.	Nsp14 (ExoN)	−49.41	−32.04	−4.56	−20.91	64.52	−80.34	28.66
21.	Nsp14 (N7mtase)	−38.83	−21.49	−4.69	−15.49	47.57	−44.45	1.51
22.	Nsp15 (Exoribonuclease)	−64.40	−48.67	−6.39	−23.20	47.29	−55.77	43.43
23.	Nsp16 (GTA site)	−63.57	−55.69	−5.17	−12.18	65.46	−62.63	8.44
24.	Nsp16 (MGP site)	−58.04	−31.42	−3.89	−22.67	59.61	−79.15	30.67
25.	Nsp16 (SAM site)	−54.88	−34.65	−4.69	−21.69	74.67	−74.43	15.52

All the energy values are given in kcal/mol; ^a^: binding-free energy; ^b^: Coulomb energy; ^c^: hydrogen-bonding correction; ^d^: lipophilic energy; ^e^: generalized Born electrostatic solvation energy; ^f^: Van der Waals energy; ^g^: ligand strain energy. ADP site: adenosine diphosphate binding site; AMP: adenosine monophosphate; C-domain: carbon-terminal domain; ExoN: N-terminal exoribonuclease; GTA: P1-7-methylguanosine-P3-adenosine-5′,5′-triphosphate; MES: 2-(N-morpholino)-ethanesulfonic acid; MGP: 7-methyl-guanosine-5′-triphosphate; N-domain: nitrogen-terminal domain; N7mtase: N7 methyltransferase; NCB site: nucleic acids binding site; N-protein: nucleocapsid protein; Nsp: non-structural protein; RBD: receptor binding domain; RdRp: RNA-dependent RNA polymerase; RNA: ribonucleic acid; RTP: remdesivir; SAM: S-adenosylmethionine.

## Data Availability

The data presented in this study are available on request from the corresponding author.

## Supplementary Materials

The following are available online at https://www.mdpi.com/article/10.3390/antibiotics10070856/s1, Figure S1: MM/GBSA optimized complexes of teicoplanin (displayed as stick) showing non-bond interactions with main protease (**A**), papain-like protease (**B**), RdRp-RTP site (**C**), RdRp-RNA site (**D**), spike protein-RBD (**E**), and spike monomer (**F**). Broken green lines enumerate hydrogen bonds whereas purple lines justify hydrophobic interactions; Figure S2. Non-bond interactions of teicoplanin (portrayed as stick style) with spike trimer (**A**), S2 protein-post fusion state (**B**), N-protein–C-domain (**C**), Nsp3-AMP site (**D**), Nsp3-MES site (**E**), and Nsp7 (**F**) in MM/GBSA optimized complexes. Hydrogen bonds are shown by broken green lines and purple lines enumerate hydrophobic interactions; Figure S3. Intermolecular interactions of teicoplanin (shown in stick style) with Nsp8 (**A**), Nsp9 (**B**), Nsp10 (**C**), Nsp12 (**D**), Nsp13 helicase-ADP site (**E**), and Nsp13 helicase-NCB site (**F**) after MM/GBSA computations. Hydrogen bonds are represented by broken green lines and purple lines specify non-polar interactions; Figure S4. Intermolecular complexes of teicoplanin (shown as sticks) with Nsp14 N-exoribonuclease (**A**), Nsp14 N7-methyl transferase (**B**), Nsp15 exoribonuclease (**C**), Nsp16-GTA site (**D**), Nsp16-MGP site (**E**), and Nsp16-SAM site (**F**) resulted after MM-GBSA analysis showing hydrogen bonds (broken green lines) and non-polar contacts (purple lines); Table S1: Intermolecular interactions observed in MM/GBSA optimized complexes of teicoplanin and 25 potential COVID-19 targets (AutoDock Vina 1.1.2 was used for molecular docking); Table S2. Computation of MM/GBSA energies of top ten docked poses of teicoplanin in complex with 25 potential COVID-19 targets.
