# Supplementary material for: Elucidation of Teicoplanin Interactions with Drug Targets Related to COVID-19
Source: Antibiotics (Basel). 2021 Jul 15;10(7):856. doi: 10.3390/antibiotics10070856 (PMC8300629; doi:10.3390/antibiotics10070856)
Supplement: Supplementary file 1 [file antibiotics-10-00856-s001.zip › antibiotics-1164502-supplementary.pdf]

Supporting Information

Elucidation of Teicoplanin Interactions with Drug Targets Related to COVID-19

Faizul Azam

Department of Pharmaceutical Chemistry & Pharmacognosy, Unaizah College of Pharmacy, Qassim University, Saudi Arabia

*Corresponding author Tel +966-50-2728652; E-mail: faizulazam@gmail.com; f.azam@qu.edu.sa

Table S1. Intermolecular interactions observed in MM/GBSA optimized complexes of teicoplanin and 25 potential COVID-19 targets (AutoDock Vina 1.1.2 was used for molecular docking).

Targets	Hydrogen bonds			Hydrophobic/Electrostatic/Other		
	Compound	Amino acid	Distance (Å)	Type	Amino acid	Distance (Å)
Main protease	H	Pro168	2.05	Alkyl	Leu141	4.97
	H	Ser46	2.21	Alkyl	Pro168	4.85
	H	Gln189	2.67	Alkyl	Leu50	4.26
	O	Met165	2.63			
	O	Gly170	3.04			
	H	Glu166	2.92			
	H	Ser46	2.96			
	H	Glu166	2.37			
Papain-like protease	O	Lys157	3.04	π-Lone Pair	Tyr264	2.89
	O	Arg166	2.88	Alkyl	Pro247	4.52
	O	Gln269	2.56			
	O	Tyr273	2.17			
	H	Tyr268	2.84			
	H	Leu162	2.26			
	H	Thr301	1.78			
	H	Glu161	1.90			
	H	Thr301	2.56			
	O	Gly163	2.60			
	O	Arg166	2.70			
	O	Tyr268	2.32			
	H	Tyr268	3.04			
	H	Gln269	2.45			
	H	Asp164	2.22			
	H	Asp164	2.46			
	H	Tyr268	2.79			
	H	Thr301	2.42			
	H	Ala246	2.78			
	H	Tyr264	2.91			
	H	Tyr268	2.69			

	O	Hie439	2.82	Alkyl	Ala547	4.18
	O	Ile548	2.47	Alkyl	Arg555	4.94
	O	Lys551	2.51	Alkyl	Val557	4.89
	O	Lys551	3.02	Alkyl	Lys551	3.92
	O	Lys551	2.07	Alkyl	Arg555	3.68
	O	Arg553	2.84	Alkyl	Val557	5.48
	O	Asn691	2.08			
	H	Glu811	2.71			
	H	U20	2.09			
	HO	Asp845	2.89			
	H	U20	1.78			
	H	Asp760	2.12			
	H	A19	2.12			
	H	His816	2.93			
	H	A19	2.57			
	H	Ser814	1.76			
RdRp (RTP site)	O	Hie439	2.21			
	O	Ala547	2.43			
	O	Lys551	2.58			
	O	Lys798	2.86			
	O	U20	2.53			
	O	A11	2.80			
	H	Asp760	2.91			
	H	A19	2.61			
	H	A19	2.86			
	H	Asp618	2.21			
	H	Asp833	2.30			
	H	U20	2.74			
	H	A19	2.76			
	H	U18	2.56			
	H	Tyr619	2.29			
	H	Ser814	2.36			
	H	Tyr619	2.37			
	O	Asn496	2.08	Alkyl	Lys500	4.09
	O	Lys500	2.90	Alkyl	Lys593	4.07
	O	Lys545	2.59	π-Alkyl	Phe594	5.23
	O	Lys545	2.75			
	O	Thr565	2.65			
	O	Arg569	2.39			
	O	Ser592	2.10			
	H	Asp865	2.80			
	HO	Gly683	1.88			
RdRp (RNA site)	H	Asp684	1.89			
	H	Tyr546	1.70			
	H	Ser501	1.87			
	H	Tyr689	1.89			
	O	Lys545	3.05			
	O	Lys545	2.71			
	O	Ala547	2.90			
	O	Ser592	2.64			
	O	Gly683	2.96			

	H	Ala685	2.80			
	H	Tyr546	3.00			
	O	Tyr453	1.94	-	-	-
	O	Gln493	2.11			
	O	Gln493	2.37			
	HO	Glu484	2.10			
Spike protein (RBD)	H	Gly485	1.84			
	H	Gln493	2.23			
	H	Ser494	2.85			
	H	Gly496	1.81			
	O	Arg403	2.49			
	H	Gln493	2.85			
	O	Tyr453	1.72	π-Alkyl	Tyr489	4.30
	O	Gly496	1.69	π-Alkyl	Tyr505	4.70
	O	Gln498	2.07			
	O	Tyr505	2.14			
Spike monomer	H	Asn501	1.73			
	H	Thr500	1.85			
	O	Tyr495	2.66			
	H	Asp405	2.57			
	H	Tyr505	2.81			
	O	Tyr170	2.01	Alkyl	Pro330	5.26
	H	Arg357	1.93	Alkyl	Ile332	5.27
	H	Glu340	2.11	π-Alkyl	Phe168	4.54
	H	Leu335	1.68			
	H	Glu169	2.68			
Spike trimer	H	Glu340	2.19			
	O	Pro337	3.01			
	H	Leu335	2.99			
	H	Asn360	2.95			
	H	Glu340	2.43			
	H	Leu335	2.72			
	H	Leu335	2.65			
	H	Asp979	2.56	Alkyl	Ala972	4.79
	H	Ser968	1.95			
S2 protein (post fusion state)	H	Asp1163	2.81			
	H	Asp1165	1.91			
	H	Asn978	2.22			
	H	Ser975	2.55			
	H	Ser968	2.72			
	O	Gln35	2.12	-	-	-
	O	Lys115	2.05			
	O	Gln35	2.16			
	O	Gln35	2.37			
	O	Arg73	2.16			
N-protein (C domain)	O	Arg73	2.83			
	O	Val78	2.94			
	H	Glu77	1.71			
	H	Met76	1.83			
	O	Gly75	2.81			
	H	Pro33	3.00			
N-protein (N domain)	O	Leu5	2.13	Alkyl	Met3	5.17

	O	Ser11	2.49	Alkyl	Pro111	5.22
	O	Arg48	2.89			
	O	Tyr69	2.39			
	O	Arg109	1.88			
	H	Asn8	1.66			
	H	Asn114	2.72			
	H	Pro111	2.76			
	H	Asn7	1.94			
	O	Ala10	3.08			
	H	Tyr71	2.38			
	H	Pro111	2.82			
	H	Leu5	2.64			
	H	Leu5	2.52			
	H	Asn7	2.56			
	O	Gly48	2.93	Alkyl	Ala38	3.76
	O	Lys163	2.73	Alkyl	Val49	4.21
	O	Lys163	2.76	Alkyl	Pro125	5.02
	H	Asp22	1.79			
	H	Asn101	1.77			
	H	Gly48	2.48			
	H	Asp22	2.10			
	H	Asp157	1.90			
	O	Gly47	2.45			
Nsp3 (AMP site)	O	Gly47	2.64			
	O	Gly48	2.63			
	O	Val49	2.27			
	O	Gly133	2.76			
	H	Gly130	2.73			
	H	Asp157	2.56			
	H	Gly46	2.52			
	H	Asp157	3.06			
	H	Asp22	2.68			
	H	Asp22	2.86			
	O	Asn40	2.94	Alkyl	Val155	4.41
	O	Gly48	2.81	Alkyl	Ile131	4.05
	HO	Gly46	1.71			
	H	Ala129	1.86			
	H	Hie45	3.05			
	H	Gly51	1.90			
Nsp3 (MES site)	O	Gly46	2.71			
	O	Gly47	2.56			
	O	Lys102	2.82			
	O	Pro136	2.79			
	H	Ala129	2.63			
	H	Gly46	2.49			
	H	Gly46	2.60			
	O	Gln31	1.79	-	-	-
	O	Ser57	2.15			
Nsp7	H	Glu50	2.97			
	H	Ser54	2.38			
	H	Gln31	2.01			

	H	Val53	2.11			
	H	Glu50	1.74			
	H	Gln31	1.80			
	O	Lys51	2.55			
	O	Lys51	2.87			
	H	Ser54	3.00			
	H	Glu50	2.87			
	H	Ser54	3.07			
Nsp8	HO	Met94	2.08	Alkyl	Pro121	3.99
	H	Met129	1.62	Alkyl	Leu117	4.31
	H	Met129	2.92	Alkyl	Pro121	4.12
	H	Thr124	1.96	Alkyl	Val131	5.02
	O	Met90	2.64	Alkyl	Pro121	4.73
	O	Lys97	2.57			
	H	Ser164	2.75			
	H	Met129	2.47			
Nsp9	O	Leu9	2.98	Alkyl	Val110	4.88
	O	Ser13	1.96			
	O	Arg39	1.92			
	HO	Thr109	2.75			
	H	Leu9	1.72			
	H	Asp78	1.84			
	H	Ser105	1.81			
	O	Met12	2.77			
	O	Asn27	2.74			
	H	Leu9	2.90			
Nsp10	O	Lys4348	2.83	Alkyl	Val4369	4.61
	O	Lys4348	2.80	Alkyl	Val4369	4.75
	H	Asn4338	2.00	π-Alkyl	Phe4342	5.46
	H	Pro4337	1.70			
	H	Ile4308	1.95			
	O	Pro4339	2.39			
	O	Pro4339	2.57			
	O	Lys4348	2.57			
	H	Pro4337	2.87			
	H	Asn4338	2.59			
	H	Pro4339	2.57			
	H	Cyt4370	2.58			
	H	Phe4342	3.02			
Nsp12	O	Hie439	1.96	Alkyl	Ala688	4.05
	O	Ala550	1.85	Alkyl	Cys813	5.42
	O	Lys551	2.29	Alkyl	Leu758	4.88
	O	Arg555	2.68	Alkyl	Cys813	3.93
	O	Lys593	2.86			
	O	Lys621	2.14			
	O	Asp623	3.00			
	O	Ala688	2.37			
	O	Asn691	2.43			
	O	Gln815	2.52			
	HO	Asp684	2.33			
	H	Asp865	2.14			

	H	Ser814	1.95			
	H	Asp760	1.92			
	O	Ser549	2.72			
	O	Lys551	2.79			
	H	Asp623	2.53			
	H	Tyr619	3.09			
	H	Tyr619	2.40			
	H	Cys813	2.71			
	H	Ser814	2.61			
	H	Asp760	2.54			
	H	Ser549	2.69			
Nsp13 (helicase ADP site)	O	Ser289	2.79	Alkyl	Ala312	3.46
	O	Lys320	2.41	Alkyl	Ala316	3.60
	O	Lys320	2.80			
	O	Lys323	2.18			
	O	Arg442	1.96			
	H	Ser539	1.67			
	H	Ala316	1.82			
	H	Tyr324	1.89			
	H	Arg442	2.16			
	H	Glu319	2.29			
	O	Ser289	2.47			
	H	Arg442	2.91			
	H	Ser289	3.02			
	H	His290	2.93			
Nsp13 (helicase NCB site)	O	Arg178	1.95	Alkyl	Ala312	4.47
	O	Gln531	3.04	Alkyl	Val340	4.04
	H	Asp534	2.53			
	HO	Glu201	1.97			
	H	Asp534	1.95			
	H	Thr530	1.79			
	H	Gln537	2.51			
	H	Ser539	3.08			
	H	Glu375	2.89			
	O	Pro529	2.74			
	O	Ser535	2.53			
	O	Ser535	2.25			
	O	Gly538	2.51			
	H	Asp534	2.38			
	H	Glu201	2.45			
	H	Gln537	2.43			
	H	Thr530	2.46			
	H	Gln537	2.95			
Nsp14 (ExoN)	O	Lys9	2.19	Alkyl	Ala187	4.09
	O	Ser56	2.15	Alkyl	Ala187	5.03
	O	Gly59	2.64			
	O	Phe60	2.84			
	O	Lys61	2.51			
	O	Leu253	2.79			
	H	Gly6	1.88			
	H	Gln245	2.44			
	H	Thr250	3.07			

	H	Gly251	1.90			
	O	Gly59	2.61			
	O	Gly59	2.69			
	O	Gly59	3.03			
	O	Gly102	2.85			
	O	Gly102	2.98			
	O	Asn252	2.36			
	H	Gly102	2.29			
	H	Gly251	2.82			
	H	Gln245	2.76			
	O	Arg310	2.15	-	-	-
	O	Gln354	2.44			
	O	Ser357	2.04			
Nsp14 (N7mtase)	HO	Arg289	1.84			
	H	Asp291	2.75			
	H	Pro355	2.99			
	H	Hie424	2.35			
	O	Lys336	2.47			
	O	Hie235	2.24	Alkyl	Cys291	3.97
	O	Hie235	2.67	π-Alkyl	Trp333	5.20
	O	His243	1.99	π-Alkyl	Trp333	4.83
	O	Ser244	1.95	π-Alkyl	Trp333	4.19
	O	Gln245	2.55	π-Alkyl	Trp333	4.61
	O	Cys291	2.19	π-Alkyl	Tyr343	3.81
	H	Gln245	1.78			
Nsp15 (Exoribonuclease)	H	His243	1.95			
	H	Ser244	1.74			
	H	Glu340	2.35			
	H	Tyr343	1.79			
	O	Hie235	2.98			
	O	Ser244	3.07			
	O	Gly247	2.96			
	H	Gln245	2.28			
	H	Glu340	2.48			
	O	Lys6935	2.29	Alkyl	Lys6935	4.79
	O	Ser6999	2.03	Alkyl	Lys6935	4.95
	H	Asp6873	2.59	π-Alkyl	Tyr6930	5.39
	H	Tyr6930	1.77			
	H	Asn6899	2.66			
	H	Tyr6828	1.92			
	H	Asp6830	2.04			
Nsp16 (GTA site)	H	Asp6928	1.90			
	O	Ser6872	2.62			
	O	Met6929	2.85			
	O	Lys6935	2.61			
	H	Asn6996	2.68			
	H	Gly6869	2.90			
	H	Asp6928	2.62			
	H	Asn6996	2.55			
Nsp16 (MGP site)	O	Ala6808	2.77	π-Alkyl	Trp6987	4.56
	H	Ser6800	1.93	π-Alkyl	Trp6987	5.00

	H	Gln6804	1.70	π-Alkyl	Trp6987	4.67
	H	Ser7074	2.29	π-Alkyl	Trp6987	4.20
	H	Ala6808	1.77			
	H	Asn7008	2.83			
	H	Ser7074	2.87			
	O	Thr6854	2.78			
	O	Thr6854	2.74			
	H	Ala6808	2.74			
	H	Thr6856	2.82			
	H	Thr6856	3.00			
	O	Tyr6930	2.78	-	-	-
	O	Lys6935	2.32			
	O	Lys6935	2.97			
	O	Lys6935	2.63			
	O	Ser6999	2.40			
	H	Asp6928	1.82			
Nsp16 (SAM site)	H	Gly6871	1.98			
	H	Asp6897	2.58			
	H	Asp6928	1.92			
	H	Asn6996	1.84			
	O	Gly6829	2.86			
	O	Gly6829	2.50			
	H	Asp6897	2.86			
	H	Asn6996	2.50			

Table S2. Computation of MM/GBSA energies of top ten docked poses of teicoplanin in complex with 25 potential COVID-19 targets.

SN	Targets	Docking Models	AutoDock Vina ΔG_{Bind}	MM/GBSA						
				ΔG_{Bind}	ΔG_{Coul}	ΔG_{HBond}	ΔG_{Lipo}	SolvGB	ΔG_{vdw}	Lig SE
1	Main protease	1M1	-5.7	-64.91	-25.07	-2.64	-49.96	41.57	-53.29	40.50
		1M2	-5.7	-46.33	-27.67	-0.73	-45.85	49.04	-51.94	44.16
		1M3	-5.4	-61.45	-10.99	-1.09	-48.02	44.53	-66.48	42.41
		1M4	-5.4	-97.55	-19.11	-0.68	-59.81	44.25	-68.46	3.87
		1M5	-5.3	-42.53	-5.12	-1.62	-29.12	34.28	-50.01	54.54
		1M6	-5.2	-32.51	-7.36	-3.87	-47.17	46.82	-52.82	71.58
		1M7	-5.2	-58.97	14.97	-1.49	-49.93	38.00	-65.00	36.50
		1M8	-5.2	-67.90	-18.75	-0.65	-53.78	47.89	-58.45	19.15
		1M9	-5.1	-46.37	-14.41	-3.08	-36.69	41.28	-48.26	53.40
		1M10	-5.1	-56.25	-30.91	-1.93	-59.75	54.17	-46.81	42.45
2	Papain-like protease	2M1	-5.4	-78.75	-39.06	-3.33	-58.73	54.34	-42.74	39.85
		2M2	-5.2	-74.17	-25.87	-2.61	-53.59	58.52	-52.61	18.25
		2M3	-5.1	-70.66	-53.54	-4.33	-43.54	56.28	-34.50	38.76
		2M4	-5.1	-47.95	-37.96	-1.05	-34.11	46.03	-41.05	40.92
		2M5	-5.1	-52.18	-10.37	-1.55	-47.00	48.32	-45.61	16.46
		2M6	-4.7	-46.55	-28.60	-0.87	-28.98	37.45	-41.19	21.59
		2M7	-4.7	-25.48	-16.71	-1.45	-35.50	45.65	-57.59	58.92
		2M8	-4.6	-59.04	-33.59	-1.33	-50.31	43.26	-59.09	62.35
		2M9	-4.6	-53.41	-31.57	-2.99	-22.03	48.41	-43.28	9.55
		2M10	-4.6	-51.19	-30.35	-2.22	-56.59	50.69	-43.79	63.80
3		3M1	-9.8	75.76	-124.58	-4.94	-91.90	209.45	-17.65	184.45

		3M2	-9.5	213.43	-48.00	-6.35	-39.95	223.55	-29.34	184.68
		3M3	-9.5	253.29	-114.47	-7.47	-35.74	239.96	-15.42	272.34
		3M4	-9.4	117.68	-117.20	-6.77	-37.75	250.73	-64.96	133.94
		3M5	-9.3	203.79	-41.73	-7.24	-24.03	243.74	-62.70	164.86
	RdRp (RTP site)	3M6	-9.2	237.92	-60.39	-6.64	-43.14	205.47	20.84	201.55
		3M7	-9.1	217.46	-63.92	-7.47	-37.20	198.79	-1.13	192.32
		3M8	-9.1	223.38	-92.89	-6.56	-39.18	208.35	-0.71	221.88
		3M9	-9	122.55	-133.38	-8.90	-42.75	293.51	-46.87	111.36
		3M10	-8.9	139.53	-135.56	-6.38	-40.16	262.03	-33.97	154.17
		4M1	-8	-80.86	-31.92	-3.56	-49.26	76.38	-93.03	47.15
		4M2	-7.9	-67.31	-64.86	-7.71	-11.69	81.61	-70.62	48.70
		4M3	-7.8	-54.12	-40.83	-4.58	-17.85	71.17	-76.88	28.45
		4M4	-7.7	-31.16	-27.12	-6.97	-18.03	68.16	-84.02	44.52
4	RdRp (RNA site)	4M5	-7.7	-65.71	-42.03	-3.42	-21.59	90.58	-103.70	16.33
		4M6	-7.7	-38.91	-32.71	-5.02	-21.65	71.14	-78.36	40.00
		4M7	-7.7	-86.33	-68.97	-6.24	-13.97	78.93	-83.45	10.54
		4M8	-7.6	-71.88	-35.76	-4.90	-25.19	57.49	-74.53	30.50
		4M9	-7.6	-63.35	-3.24	-4.95	-22.01	53.50	-88.85	7.60
		4M10	-7.5	-46.32	-29.49	-5.00	-15.19	75.62	-86.77	17.43
		5M1	-6.5	-74.61	-31.09	-4.78	-46.45	32.98	-53.94	30.35
		5M2	-6.3	-91.99	-40.79	-1.98	-46.65	45.40	-51.99	21.37
		5M3	-6.3	-42.47	-32.73	-1.28	-26.75	43.82	-56.38	47.64
		5M4	-6.3	-59.41	-35.06	-2.61	-26.04	50.50	-54.06	31.24
5	Spike protein (RBD)	5M5	-6.2	-61.83	-31.56	-2.27	-44.14	49.98	-54.09	58.67
		5M6	-6.2	-82.43	-40.93	-2.43	-46.19	48.65	-52.83	33.67
		5M7	-6.2	-73.19	-62.56	-4.00	-38.54	52.55	-45.98	21.05
		5M8	-6.1	-95.76	-32.67	-2.13	-54.25	48.60	-66.71	18.19
		5M9	-6	-48.85	-28.39	-1.24	-17.57	43.40	-58.68	38.99
		5M10	-5.9	-37.69	-21.65	-4.11	-29.59	36.16	-51.90	68.18
		6M1	-5.9	-66.37	-28.83	-2.87	-33.02	41.91	-53.45	23.41
		6M2	-5.7	-60.41	-20.71	-3.46	-21.67	49.85	-72.35	10.47
		6M3	-5.5	-42.80	-28.25	-4.88	-15.01	46.75	-53.37	29.61
		6M4	-5.4	-68.38	-61.51	-4.39	-13.35	55.98	-50.51	12.74
6	Spike mono-mer	6M5	-5.3	-60.45	-35.63	-4.99	-16.62	52.30	-61.41	32.42
		6M6	-5.1	-39.04	-37.69	-5.51	-14.41	43.93	-41.89	40.92
		6M7	-5.1	-32.77	-6.19	-5.53	-15.28	40.33	-61.59	37.39
		6M8	-4.9	-33.54	-33.91	-4.65	-14.65	49.13	-57.05	35.52
		6M9	-4.9	-52.97	-39.13	-5.16	-14.41	30.19	-35.12	18.00
		6M10	-4.9	-21.68	-26.69	-2.91	-12.79	44.51	-39.90	19.71
		7M1	-7	-45.17	-31.10	-3.69	-26.16	52.35	-51.26	50.09
		7M2	-7	-40.62	-24.38	-2.45	-27.74	52.50	-64.60	30.83
		7M3	-6.9	-66.87	-27.37	-4.11	-21.53	53.95	-65.20	11.90
		7M4	-6.8	-58.88	-25.84	-4.74	-23.74	43.18	-60.36	36.34
7	Spike trimer	7M5	-6.8	-30.88	-7.72	-3.99	-22.06	45.59	-65.77	51.27
		7M6	-6.7	-36.28	-20.40	-3.06	-19.38	44.04	-70.13	38.92
		7M7	-6.7	-39.74	-30.06	-2.55	-15.91	55.91	-58.96	19.47
		7M8	-6.7	-14.41	-16.83	-4.29	-23.21	64.93	-70.51	61.70
		7M9	-6.6	-46.41	-1.90	-2.85	-27.63	57.14	-81.01	23.31
		7M10	-6.6	-43.74	-28.36	-2.84	-17.77	48.76	-58.50	20.65
		8M1	-5.7	-25.01	-22.12	-3.50	-11.40	61.44	-70.72	30.74
8	S2 protein (post fusion state)	8M2	-5.6	-16.94	-19.57	-3.37	-10.79	49.75	-59.71	29.01
		8M3	-5.5	-34.28	-20.12	-3.33	-14.41	56.83	-63.82	16.74

		8M4	-5.3	-36.93	-23.22	-2.74	-14.11	55.19	-61.27	24.45
		8M5	-5.2	-13.34	-2.29	-0.45	-12.66	44.62	-57.77	31.85
		8M6	-5.2	-8.02	-45.43	-2.78	-13.00	96.04	-60.31	27.81
		8M7	-5.1	-21.95	-21.01	-2.64	-14.25	57.36	-66.30	41.41
		8M8	-5	-8.93	-43.83	-3.07	-12.01	99.61	-56.45	27.36
		8M9	-4.9	-29.72	-45.05	-3.26	-14.98	83.85	-61.54	12.77
		8M10	-4.9	-14.18	-20.44	-2.53	-15.49	79.39	-70.49	29.54
9	N-protein (C domain)	9M1	-6.1	-34.14	-17.41	-3.14	-18.18	62.01	-63.42	17.77
		9M2	-6.1	-22.10	-38.33	-3.49	-9.96	64.64	-50.20	36.01
		9M3	-5.7	-35.76	-33.37	-4.27	-18.96	69.74	-68.03	29.21
		9M4	-5.7	-30.45	-9.78	-4.67	-19.99	48.32	-67.16	38.30
		9M5	-5.6	-42.31	-30.82	-5.06	-16.05	64.88	-62.43	26.93
		9M6	-5.6	-28.79	1.21	-2.26	-18.55	38.59	-55.07	19.45
		9M7	-5.5	-13.17	-4.55	-2.66	-25.30	66.29	-69.88	37.08
		9M8	-5.5	-20.37	-9.70	-1.67	-21.23	41.66	-51.36	34.39
		9M9	-5.4	-40.57	-33.98	-3.22	-17.73	62.10	-58.74	26.57
		9M10	-5.3	-20.59	-13.97	-2.84	-16.50	37.59	-50.91	46.02
10	N-protein (N domain)	10M1	-8.8	-88.69	-38.01	-5.70	-19.15	47.39	-74.01	14.84
		10M2	-8.7	-64.99	-14.73	-4.54	-19.23	44.95	-73.27	29.76
		10M3	-8.5	-51.02	-20.97	-4.87	-24.51	48.77	-73.49	50.30
		10M4	-8.4	-43.37	-31.79	-4.45	-23.05	53.94	-67.14	39.75
		10M5	-8.2	-39.70	-25.53	-4.80	-30.34	58.62	-78.82	59.51
		10M6	-7.9	-45.23	-26.57	-5.48	-25.33	52.24	-66.46	43.61
		10M7	-7.9	-43.37	-39.45	-5.68	-9.95	61.56	-61.00	52.34
		10M8	-7.8	-53.25	-23.28	-4.22	-16.99	44.70	-70.46	47.13
		10M9	-7.7	-13.19	-20.61	-5.13	-10.61	61.16	-68.57	80.32
		10M10	-7.4	-102.13	-40.95	-6.98	-24.95	61.07	-78.69	-0.90
11	Nsp3 (AMP site)	11M1	-7.3	-37.74	1.62	-2.22	-28.83	39.04	-67.60	36.07
		11M2	-7.3	-58.33	-42.82	-4.38	-25.86	64.42	-71.09	32.93
		11M3	-7.3	-34.25	-2.31	-2.27	-25.57	31.96	-65.18	30.90
		11M4	-7.1	-44.98	-17.89	-2.56	-25.49	52.30	-70.59	35.64
		11M5	-6.6	-26.80	9.51	-0.47	-21.45	25.16	-60.25	28.75
		11M6	-6.6	-27.10	-2.74	-1.44	-17.34	45.46	-67.53	44.71
		11M7	-6.6	-49.22	-31.83	-3.35	-16.14	51.02	-70.27	25.62
		11M8	-6.4	-23.60	-18.81	-2.37	-15.06	47.09	-53.49	43.34
		11M9	-6.4	-54.41	-11.12	-2.79	-17.08	33.56	-65.77	32.16
		11M10	-6.3	-54.16	-17.77	-2.30	-12.49	39.34	-65.03	3.82
12	Nsp3 (MES site)	12M1	-6.1	-43.26	-10.27	-3.06	-24.41	49.16	-67.74	36.28
		12M2	-6.1	-33.82	-14.29	-4.56	-28.21	46.29	-56.82	61.07
		12M3	-5.9	-57.60	-12.61	-2.42	-30.13	34.62	-75.40	37.34
		12M4	-5.6	-47.68	-32.35	-3.44	-18.10	49.46	-61.22	33.35
		12M5	-5.5	-51.61	-17.26	-3.03	-21.12	43.16	-61.40	26.87
		12M6	-5.4	-42.97	-11.89	-1.94	-26.85	34.94	-62.88	38.08
		12M7	-5.3	-43.63	-27.19	-2.99	-22.22	40.83	-50.56	28.24
		12M8	-5.2	-48.37	-29.98	-4.36	-25.62	42.04	-52.37	39.95
		12M9	-5.2	-36.15	-25.57	-4.74	-15.39	35.39	-47.63	38.73
		12M10	-5.1	-43.33	-3.25	-1.54	-20.34	30.26	-65.38	25.17
13	Nsp7	13M1	-5.2	-26.63	-25.65	-1.50	-13.13	50.89	-50.83	18.66
		13M2	-4.9	-42.29	-34.84	-2.81	-19.73	56.40	-58.37	18.41
		13M3	-4.7	-7.18	-5.28	-3.28	-13.07	46.38	-51.46	52.87
		13M4	-4.4	-66.61	-54.39	-4.15	-19.17	55.49	-60.78	8.43
		13M5	-4.1	-19.64	-29.42	-3.42	-12.66	47.18	-45.67	40.05

		13M6	-3.9	-4.02	2.55	-1.97	-11.15	37.51	-58.47	46.14
		13M7	-3.9	-32.59	-16.95	-2.77	-14.77	45.27	-47.03	13.64
		13M8	-3.9	-44.20	-35.42	-3.37	-21.33	66.94	-65.69	22.09
		13M9	-3.8	-22.57	-15.52	-3.78	-14.77	46.48	-57.15	34.62
		13M10	-3.7	-21.10	-31.49	-3.31	-17.03	54.38	-60.19	49.76
14	Nsp8	14M1	-7	-40.86	8.45	-1.97	-27.19	24.42	-68.20	44.83
		14M2	-6.7	-10.97	21.42	-1.77	-14.58	28.08	-62.69	58.04
		14M3	-6.2	-64.46	-19.68	-3.01	-27.43	34.13	-54.25	31.30
		14M4	-6.2	-32.48	9.13	-1.54	-20.01	30.57	-57.92	38.90
		14M5	-6.1	-43.84	3.97	-0.85	-21.39	31.97	-63.54	29.91
		14M6	-6.1	-58.54	-1.60	-3.21	-23.07	23.68	-68.72	35.54
		14M7	-6	-56.07	-3.84	-1.92	-30.28	21.53	-59.84	29.46
		14M8	-5.9	-67.93	-10.55	-2.28	-25.51	28.75	-68.85	16.42
		14M9	-5.9	-9.80	-0.87	-1.50	-21.33	34.71	-46.76	48.31
		14M10	-5.9	-57.18	-23.05	-2.39	-22.58	42.24	-67.22	29.99
15	Nsp9	15M1	-5	-65.41	-44.21	-5.05	-20.94	61.63	-68.43	26.18
		15M2	-4.6	-29.70	-12.43	-3.27	-15.18	52.94	-66.93	26.23
		15M3	-4.5	-66.83	-43.87	-3.59	-19.55	46.75	-57.02	10.15
		15M4	-4.3	0.66	5.02	-1.54	-7.59	29.09	-46.72	41.15
		15M5	-4	-58.92	-33.40	-3.73	-17.80	55.27	-67.12	10.53
		15M6	-3.8	-22.74	-31.88	-2.38	-12.94	54.98	-45.30	29.37
		15M7	-3.4	-32.33	-34.35	-3.32	-13.73	46.62	-48.05	40.14
		15M8	-3.4	-24.26	-19.53	-2.80	-11.71	49.97	-59.36	39.80
		15M9	-3.3	-35.83	-8.80	-2.65	-17.22	49.54	-63.28	25.47
		15M10	-3.1	-25.58	-13.79	-3.74	-9.18	42.58	-51.15	32.35
16	Nsp10	16M1	-4.4	-11.29	-5.33	-4.47	-16.23	61.45	-60.16	56.98
		16M2	-2.8	-16.01	-15.59	-3.35	-9.32	54.52	-52.29	13.19
		16M3	-2.6	-25.83	-30.15	-4.77	-11.85	54.10	-53.85	40.00
		16M4	-2.3	-35.50	-21.16	-3.83	-11.13	44.07	-52.46	18.57
		16M5	-2.2	-47.56	-29.62	-2.47	-23.97	57.87	-62.53	40.47
		16M6	-1.7	-34.50	-42.90	-2.76	-12.47	54.17	-43.17	9.41
		16M7	-1.6	-22.69	-19.80	-3.29	-20.89	56.23	-53.17	40.06
		16M8	-1.6	-31.33	-7.44	-0.60	-13.64	35.27	-54.00	0.49
		16M9	-1.4	-13.16	-24.80	-4.55	-0.46	56.80	-39.61	32.66
		16M10	-1.1	-44.10	-27.82	-2.80	-11.34	45.56	-52.31	9.99
17	Nsp12	17M1	-8	-69.05	-39.13	-6.34	-18.19	79.99	-102.58	36.33
		17M2	-8	-68.41	-46.76	-5.99	-23.37	89.68	-106.35	35.49
		17M3	-7.8	-87.84	-96.33	-8.48	-19.26	135.75	-108.20	8.52
		17M4	-7.4	-11.32	-40.30	-4.84	-19.48	109.74	-94.39	50.76
		17M5	-7.2	-24.57	-49.55	-7.52	-23.55	126.72	-90.20	52.20
		17M6	-7.1	-8.66	-45.04	-6.20	-22.82	96.19	-69.71	49.76
		17M7	-7.1	23.00	-15.87	-5.08	-9.41	74.06	-52.53	62.29
		17M8	-7.1	8.76	-25.15	-6.14	-19.96	91.35	-63.61	54.27
		17M9	-7	-34.11	-29.02	-8.72	-12.57	67.09	-76.06	47.50
		17M10	-7	67.78	-21.66	-5.26	-17.41	102.26	-45.75	83.32
18	Nsp13 (helicase ADP site)	18M1	-6.6	10.59	-48.06	-6.21	-14.26	89.32	-41.34	54.40
		18M2	-6.6	2.60	-30.10	-5.03	-14.48	68.84	-51.54	42.07
		18M3	-6.5	-41.57	-63.94	-7.00	-19.36	88.70	-53.79	16.23
		18M4	-6.4	-0.80	-10.63	-3.93	-12.45	54.81	-46.29	41.87
		18M5	-6.2	-29.82	-2.92	-3.60	-8.18	44.81	-62.99	13.90
		18M6	-6.2	-20.65	-38.92	-4.12	-12.96	69.06	-58.74	26.05
		18M7	-6.2	-22.47	-54.76	-6.55	-5.06	78.91	-43.44	39.00

		18M8	-6.1	1.47	-15.95	-6.03	-13.33	69.21	-51.47	27.64
		18M9	-6	-35.99	-36.54	-2.79	-12.01	65.42	-57.06	12.82
		18M10	-5.9	-16.45	-15.80	-1.71	-8.68	54.10	-48.38	15.65
19	Nsp13 (helicase NCB site)	19M1	-6.4	-20.11	-50.92	-4.46	-23.73	92.54	-62.00	40.26
		19M2	-6.4	0.74	-37.44	-4.95	-26.35	95.39	-75.95	67.99
		19M3	-6.4	-13.41	-45.41	-6.32	-20.53	75.40	-46.06	52.14
		19M4	-6.2	68.15	-46.42	-5.18	-28.20	109.29	-39.46	96.50
		19M5	-6.2	-47.55	-66.12	-6.54	-23.12	118.38	-88.16	39.58
		19M6	-6.2	5.63	-53.23	-5.76	-29.98	108.00	-58.60	60.71
		19M7	-5.8	-7.70	-34.37	-5.17	-31.20	97.76	-80.37	59.34
		19M8	-5.8	38.83	-20.52	-5.42	-10.76	81.06	-37.82	86.41
		19M9	-5.4	1.18	-28.99	-6.39	-22.58	107.15	-89.89	72.11
		19M10	-5.4	-53.99	-42.21	-4.05	-26.40	87.73	-78.57	7.39
20	Nsp14 (ExoN)	20M1	-7.7	-42.32	-30.48	-5.45	-21.60	75.61	-69.32	35.82
		20M2	-7.5	-34.92	-13.94	-3.89	-21.66	65.02	-72.26	26.70
		20M3	-7.2	-21.01	-45.14	-4.11	-20.07	96.24	-74.89	46.00
		20M4	-7.1	-49.41	-32.04	-4.56	-20.91	64.52	-80.34	28.66
		20M5	-7.1	-36.77	-17.60	-3.11	-19.90	48.46	-66.01	28.16
		20M6	-7.1	-22.00	-23.15	-4.84	-14.02	59.35	-57.56	43.03
		20M7	-7	-31.05	-1.35	-3.48	-23.24	59.98	-83.91	30.18
		20M8	-7	-20.73	-29.69	-5.58	-12.41	72.45	-70.50	49.47
		20M9	-7	-32.55	-29.75	-3.85	-15.35	61.67	-57.88	25.24
		20M10	-6.8	-46.01	-45.55	-5.37	-16.52	85.23	-74.48	25.38
21	Nsp14 (N7mtase)	21M1	-3.5	5.75	-30.70	-5.41	-9.54	69.16	-43.23	58.57
		21M2	-3.1	-38.04	-33.66	-5.47	-18.33	58.77	-48.87	11.98
		21M3	-3	-38.83	-21.49	-4.69	-15.49	47.57	-44.45	1.51
		21M4	-3	47.17	-4.40	-3.33	-31.26	83.37	-33.08	65.62
		21M5	-2.9	5.80	-25.73	-4.61	-15.36	84.18	-61.67	51.06
		21M6	-2.8	41.53	-27.06	-3.52	-31.61	82.10	-32.35	74.56
		21M7	-2.7	50.56	-19.24	-4.96	-10.93	55.34	-11.77	84.78
		21M8	-2.7	35.99	-9.50	-3.58	-14.53	66.28	-56.46	94.42
		21M9	-2.5	-29.13	-40.06	-3.58	-19.41	64.24	-46.76	27.06
		21M10	-2.3	37.93	-29.19	-3.74	-30.66	86.65	-30.51	70.73
22	Nsp15 (Exoribonuclease)	22M1	-6.9	-1.36	-20.15	-3.34	-24.02	55.24	-56.15	68.84
		22M2	-6.5	-64.40	-48.67	-6.39	-23.20	47.29	-55.77	43.43
		22M3	-6.5	-30.47	-27.83	-4.04	-16.11	53.97	-63.06	55.29
		22M4	-6.2	-50.93	-31.21	-4.28	-24.49	53.94	-61.55	34.98
		22M5	-6.2	-36.69	-17.40	-2.31	-20.42	47.15	-57.56	15.21
		22M6	-6.2	-51.59	-17.90	-4.66	-16.32	38.59	-51.78	33.42
		22M7	-6.2	-24.27	-9.37	-3.55	-16.98	41.12	-49.74	36.83
		22M8	-6.1	-42.00	-17.19	-2.07	-24.85	54.74	-68.53	24.68
		22M9	-6.1	-32.71	-20.04	-2.52	-18.38	53.65	-60.22	24.93
		22M10	-6.1	-28.17	-13.10	-2.82	-20.57	50.66	-67.83	37.73
23	Nsp16 (GTA site)	23M1	-6.8	-29.16	-33.10	-6.55	-14.60	66.25	-59.58	37.56
		23M2	-6.8	-38.00	-39.20	-6.48	-15.13	71.97	-66.32	32.38
		23M3	-6.7	-61.01	-49.28	-5.73	-17.72	70.05	-72.02	11.41
		23M4	-6.6	-42.54	-34.27	-6.25	-16.08	59.88	-65.91	19.52
		23M5	-6.4	-39.88	-33.92	-4.20	-12.48	62.05	-69.89	24.58
		23M6	-6.4	-39.16	-38.61	-4.72	-13.99	68.11	-68.73	30.44
		23M7	-6.3	-23.60	-11.17	-4.25	-12.17	49.51	-59.45	41.12
		23M8	-6.2	-63.57	-55.69	-5.17	-12.18	65.46	-62.63	8.44
		23M9	-6.1	6.44	-11.41	-2.63	-14.96	36.65	-50.62	61.06

		23M10	-6.1	-2.47	-5.32	-3.15	-10.65	49.08	-61.57	54.52
24	Nsp16 (MGP site)	24M1	-7.8	-29.24	-23.42	-3.94	-29.01	76.00	-72.54	39.15
		24M2	-7.6	-50.73	-16.98	-2.81	-31.66	58.93	-79.64	34.11
		24M3	-7.6	-33.03	-19.24	-1.91	-23.32	64.57	-74.99	27.54
		24M4	-7.5	-56.44	-45.38	-2.03	-22.08	64.42	-70.91	15.40
		24M5	-7.4	-51.13	-41.60	-1.88	-23.11	70.23	-65.10	17.51
		24M6	-7.4	-58.04	-31.42	-3.89	-22.67	59.61	-79.15	30.67
		24M7	-7.4	-49.36	-35.59	-3.11	-17.92	59.18	-63.75	22.72
		24M8	-7.3	-21.15	-19.03	-3.79	-22.04	50.95	-52.36	29.91
		24M9	-7.3	-50.94	-31.21	-1.90	-24.62	72.08	-77.65	14.32
		24M10	-7.3	-52.64	-42.04	-3.38	-22.67	61.26	-72.16	28.20
25	Nsp16 (SAM site)	25M1	-6.5	-54.88	-34.65	-4.69	-21.69	74.67	-74.43	15.52
		25M2	-6.3	-18.25	-32.58	-4.97	-22.72	73.90	-62.01	41.63
		25M3	-6	-38.31	-39.18	-5.27	-21.22	84.23	-72.94	36.95
		25M4	-5.8	-33.63	-38.97	-3.81	-10.99	59.25	-57.26	27.18
		25M5	-5.7	-41.14	-54.00	-4.02	-12.58	71.47	-57.64	21.71
		25M6	-5.7	-3.99	8.06	-2.61	-8.92	43.72	-64.09	52.14
		25M7	-5.7	-36.30	-20.54	-3.35	-13.22	46.06	-59.81	16.72
		25M8	-5.6	-10.92	-27.47	-2.55	-18.62	52.91	-33.00	25.68
		25M9	-5.6	-10.55	-21.28	-3.22	-20.16	56.94	-43.15	27.36
		25M10	-5.6	11.03	-9.91	-3.45	-18.12	63.59	-42.51	58.90

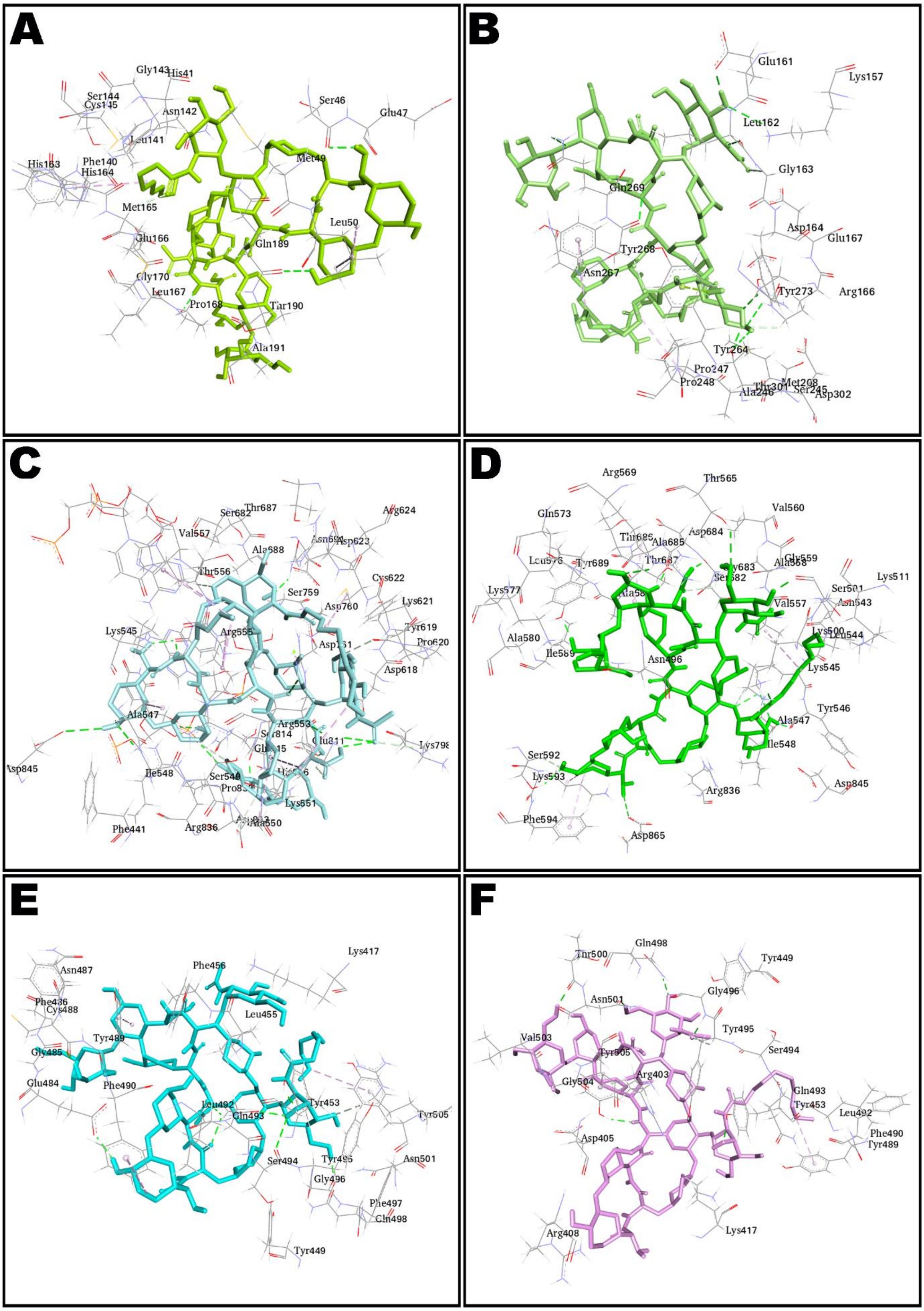

Figure S1. MM/GBSA optimized complexes of teicoplanin (displayed as stick) showing non-bond interactions with main protease (A), papain-like protease (B), RdRp-RTP site (C), RdRp-RNA site (D), spike protein-RBD (E), and spike monomer (F). Broken green lines enumerate hydrogen bonds whereas purple lines justify hydrophobic interactions.

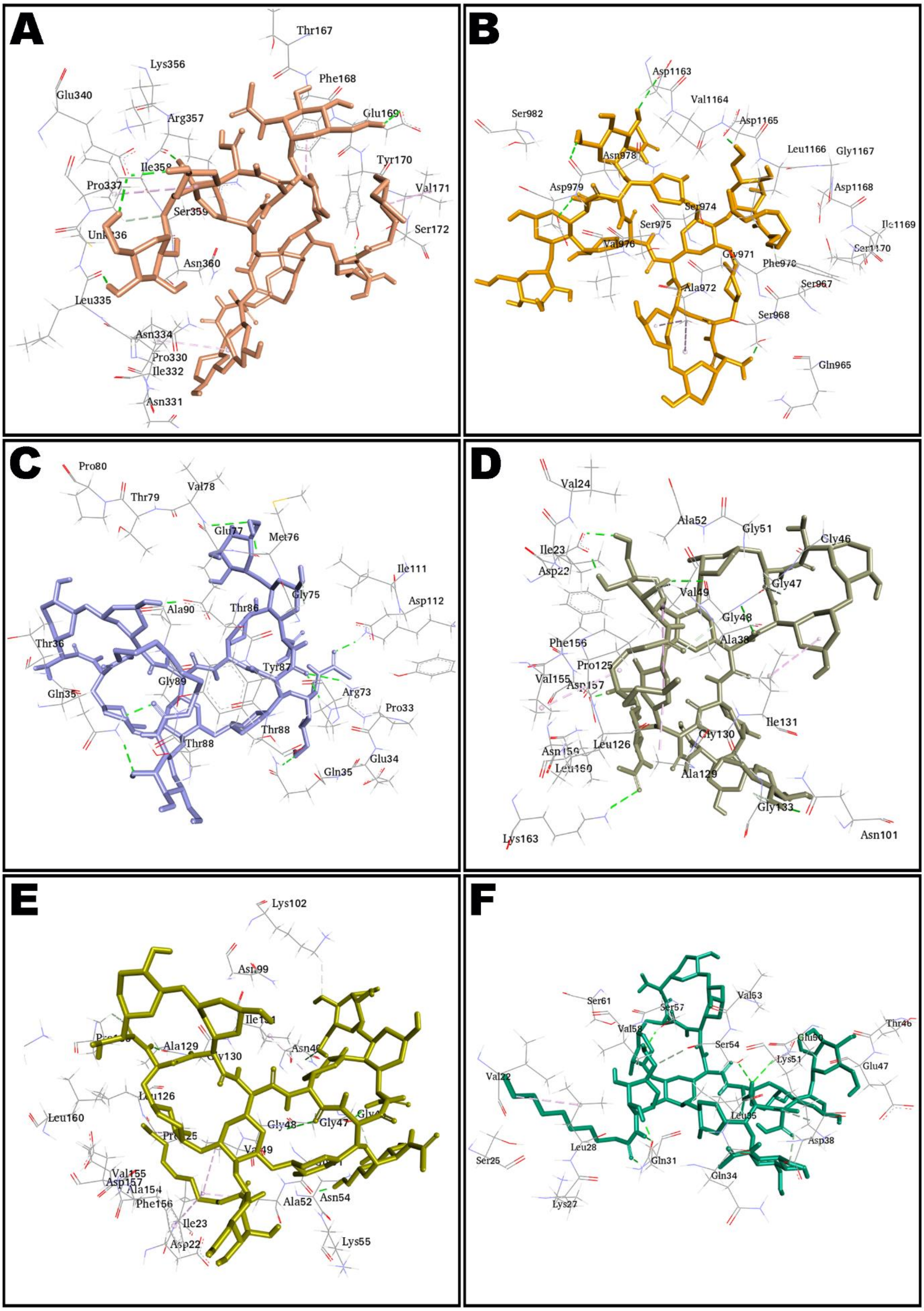

Figure S2. Non-bond interactions of teicoplanin (portrayed as stick style) with spike trimer (A), S2 protein-post fusion state (B), N-protein-C domain (C), Nsp3-AMP site (D), Nsp3-MES site (E), and Nsp7 (E) in MM/GBSA optimized complexes. Hydrogen bonds are shown by broken green lines and purple lines enumerate hydrophobic interactions.

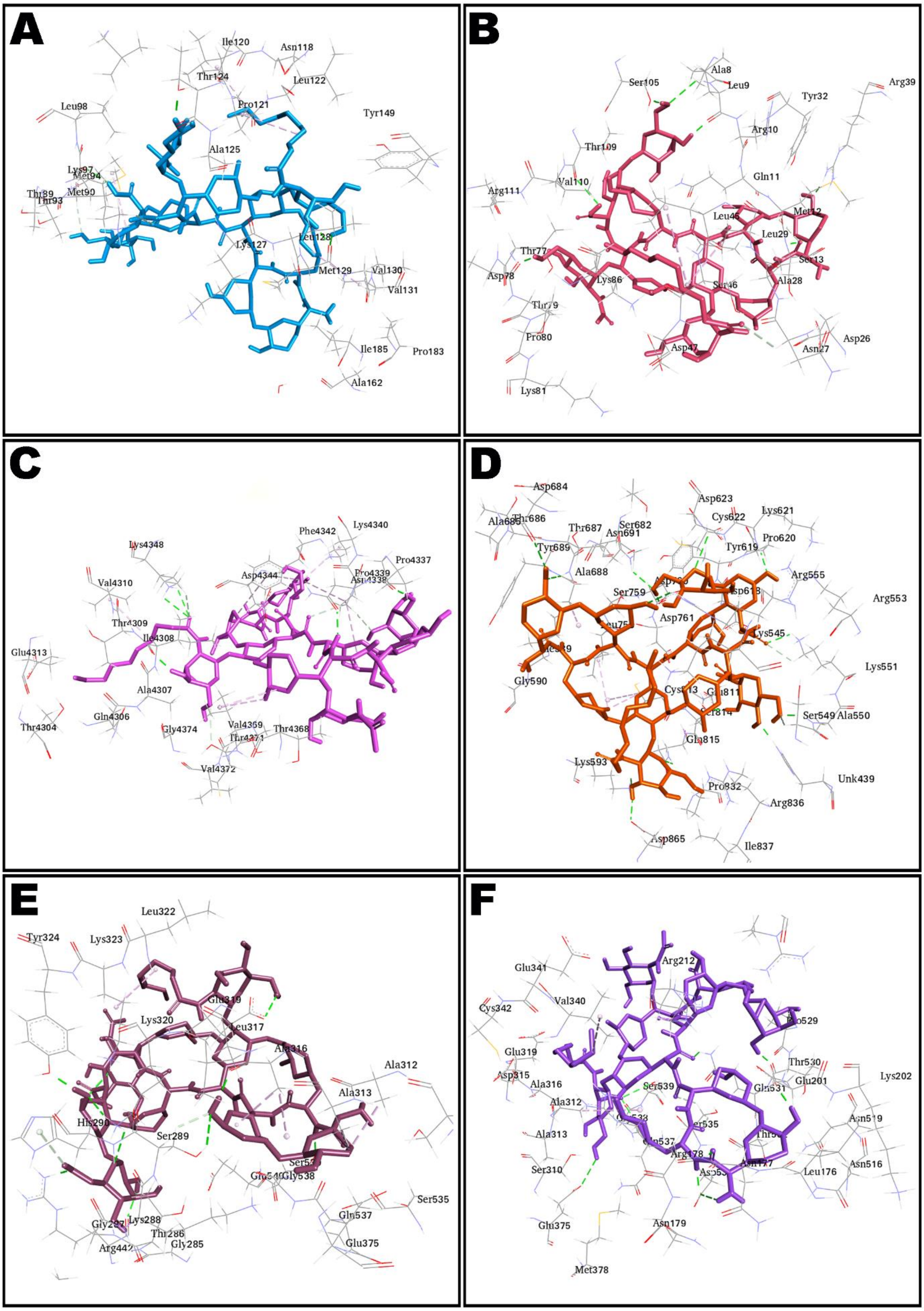

Figure S3. Intermolecular interactions of teicoplanin (shown in stick style) with Nsp8 (A), Nsp9 (B), Nsp10 (C), Nsp12 (D), Nsp13 helicase-ADP site (E), and Nsp13 helicase-NCB site (E) after MM/GBSA computations. Hydrogen bonds are represented by broken green lines and purple lines specify non-polar interactions.

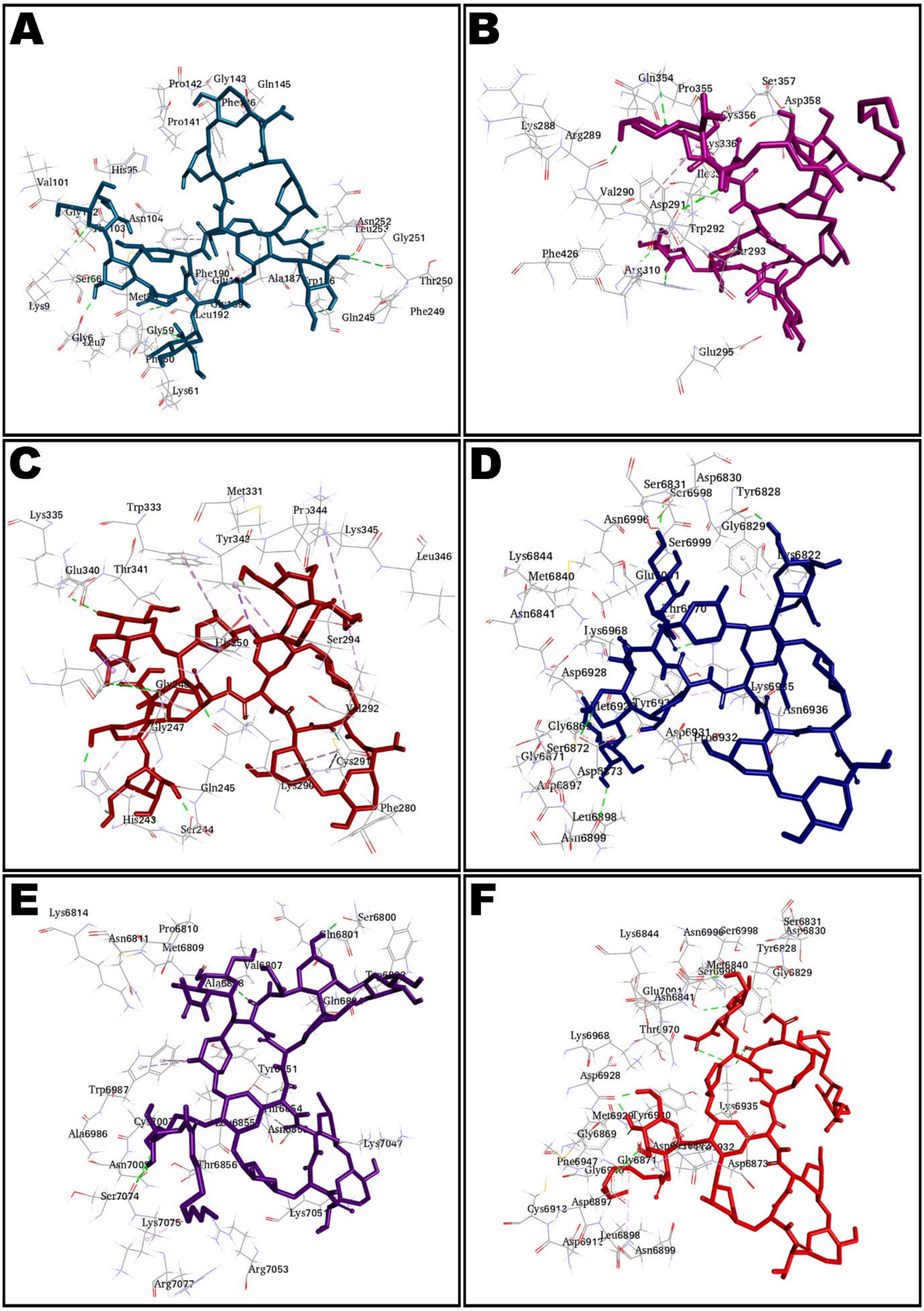

Figure S4. Intermolecular complexes of teicoplanin (shown as sticks) with Nsp14 N-exoribonuclease (A), Nsp14 N7-methyl transferase (B), Nsp15 exoribonuclease (C), Nsp16-GTA site (D), Nsp16-MGP site (E), and Nsp16-SAM site (E) resulted after MM-GBSA analysis showing hydrogen bonds (broken green lines) and non-polar contacts (purple lines).
